# A pan-cancer analysis revealing the role of LFNG, MFNG and RFNG in tumor prognosis and microenvironment

**DOI:** 10.1186/s12885-023-11545-3

**Published:** 2023-11-06

**Authors:** Xun Gong, Chenglong Zheng, Haiying Jia, Yangruiyu Liu, Rui Yang, Zizhou Chen, Yihang Pan, Xiaowu Li, Yuchen Liu

**Affiliations:** 1 Department of Hepatobiliary Surgery, Shenzhen Key Laboratory, Guangdong Provincial Key Laboratory of Regional Immunity and Diseases, International Cancer Center, Shenzhen University General Hospital, Shenzhen University Clinical Medical Academy, Shenzhen University, 1098 Xueyuan Avenue, Nanshan District, Shenzhen, 518000 Guangdong P.R. China; 2 Scientific Research Center, The Seventh Affiliated Hospital, Sun Yat-sen University, Shenzhen, 518107 Guangdong China; 3 Big Data Center, The Seventh Affiliated Hospital, Sun Yat-sen University, Shenzhen, 518107 Guangdong China

**Keywords:** Pan-cancer analysis, Fringe, Glycosyltransferases, Prognosis, Invasion and Metastasis

## Abstract

**Background:**

Fringe is a glycosyltransferase involved in tumor occurrence and metastasis. However, a comprehensive analysis of the Fringe family members lunatic fringe (LFNG), manic fringe (MFNG), radical fringe (RFNG) in human cancers is lacking.

**Methods:**

In this study, we performed a pan-cancer analysis of Fringe family members in 33 cancer types with transcriptomic, genomic, methylation data from The Cancer Genome Atlas (TCGA) project. The correlation between Fringe family member expression and patient overall survival, copy number variation, methylation, Gene Ontology enrichment, and tumor-infiltrating lymphocytes (TILs) was investigated by using multiple databases, such as cBioPortal, Human Protein Atlas, GeneCards, STRING, MSigDB, TISIDB, and TIMER2. In *vitro* experiments and immunohistochemical assays were performed to validate our findings.

**Results:**

High expression levels of LFNG, MFNG, RFNG were closely associated with poor overall survival in multiple cancers, particularly in pancreatic adenocarcinoma (PAAD), uveal melanoma (UVM), and brain lower-grade glioma (LGG). Copy number variation analysis revealed that diploid and gain mutations of LFNG was significantly increased in PAAD and stomach adenocarcinoma (STAD), and significantly associated with the methylation levels in promoter regions. Significant differential genes between high and low expression groups of these Fringe family members were found to be consistently enriched in immune response and T cell activation pathway, extracellular matrix adhesion pathway, RNA splicing and ion transport pathways. Correlation between the abundance of tumor-infiltrating lymphocytes (TILs) and LFNG, MFNG, and RFNG expression showed that high LFNG expression was associated with lower TIL levels, particularly in PAAD. In vitro experiment by using pancreatic cancer PANC1 cells showed that LFNG overexpression promoted cell proliferation and invasion. Immunohistochemical assay in 90 PAAD patients verified the expression level of LFNG and its relationship with the prognosis.

**Conclusions:**

Our study provides a relatively comprehensive understanding of the expression, mutation, copy number, promoter methylation level changes along with prognosis values of LFNG, MFNG, and RFNG in different tumors. High LFNG expression may serve as a poor prognosis molecular marker for PAAD.

**Supplementary Information:**

The online version contains supplementary material available at 10.1186/s12885-023-11545-3.

## Introduction

Glycosylation is a crucial post-translational modification that plays a significant role in all stages of tumor development, including tumorigenesis and metastasis [1]. Targeted glycosylation has emerged as a promising anti-tumor strategy [2]. Several aberrant glycosyltransferases, such as N-acetylglucosamine transferase III (Gn-TIII) and galactosyl transferase (GAT), have been identified as important tumor markers [3]. However, further studies are needed to elucidate the panorama insights in glycosylated proteins which regulate tumor growth and metastasis. Therefore, investigating the clinical prognosis value and cellular function of glycosylation-related genes using The Cancer Genome Atlas (TCGA), Gene Expression Omnibus (GEO) databases and in vitro cell expreriment were essential.

The Fringe family of glycosyltransferases, consisting of three members, i.e. lunatic fringe (LFNG), manic fringe (MFNG), and radical fringe (RFNG), that is closely associated with cancer progression [4]. Studies have shown that Fringe members add GlcNAc to the O-fucosylated EGF-like domains of the Notch receptor, thereby altering the Notch-Delta/Jagged affinity and modulating Notch signaling [5]. However, the Fringe members regulate Notch signaling differently; LFNG and MFNG enhance DELTA1 binding and repress JAGGED1 binding to NOTCH1, while RFNG stimulates the binding of both DELTA1 and JAGGED1 [6]. As known that Notch signaling is a crucial cell signaling pathway, as it regulates cell fate, proliferation, differentiation, and apoptosis [7]. Fringes are glycosyltransferases that transfer N-acetylglucosamine to the O-linked fucose of Notch receptors, regulated the Notch signaling activity that drives tumor formation and progression, resulting in poor prognosis [8]. Secondly, Fringes included three members LFNG, MFNG and RFNG, they have different activities toward O-fucosylated EGF repeats [9]. Although a particular Fringe member could act as a tumor suppressor in one cancer type, it may act as an oncogene in another. Thus, the specific tumor-promoting role of Fringes differs depending on the type of cancer [10]. Thirdly, they also play an important role in immune system, loss of LFNG affects thymic T cell development, and LFNG and MFNG are required for marginal zone (MZ) B cell development [11]. Therefore, the crucial functions of Fringes continue to emerge as more mechanistic studies are being pursued, further research is needed to explore their roles and therapeutic benefits in various malignancies. However, it has been revealed that Fringe members may play a dual function depending on the cancer type. studies have demonstrated that LFNG acts as a tumor suppressor in breast cancer, prostate cancer, and cervical cancer [12–14]. High levels of LFNG expression have been found to promote liver cancer progression and predict a poor prognosis [15], as well as colorectal cancer and lung cancer development [16, 17]. On the other hand, MFNG-mediated inhibition of the JAG1-NOTCH1 signaling pathway has been shown to be crucial in suppressing colorectal cancer progression and associated with a worse prognosis [18]. In contrast, high levels of MFNG expression activate Notch signaling, leading to a claudin-low breast cancer (CLBC) phenotype and promoting kidney cancer development [19]. The role of LFNG in pancreatic cancer is still controversial. Studies have shown that LFNG deficiency accelerates KRAS-induced pancreatic cancer development in mice [20], while LFNG expression inhibition suppresses pancreatic cancer cell proliferation and migration by inhibiting NOTCH1 activation [21]. These findings indicate that LFNG and MFNG can function as either tumor suppressors or tumor drivers depending on the genetic context, potentially due to differences in ligand expression in various tissues [22].

Recent studies have suggested that the Fringe family members are crucial in tumorigenesis. However, the role of LFNG, MFNG, and RFNG in human cancer remains poorly understood. Here, we conducted a pan-cancer analysis using the TCGA database to investigate the potential role of LFNG, MFNG, and RFNG in cancer. We performed the immunohistochemical assay and in vitro experiment to verify that higher expression of LFNG related to bad prognosis in pancreatic cancer patients and LFNG overexpression promoted cell proliferation and invasion. We also examined various factors, including gene expression, survival status, DNA methylation, genetic alterations, immune infiltration, and relevant cellular pathways, to elucidate the possible molecular mechanisms underlying the involvement of LFNG, MFNG, and RFNG in the pathogenesis or clinical prognosis of different cancers.

## Materials and methods

### Data collection and process

We obtained pan-cancer sequencing data from The Cancer Genome Atlas (TCGA) portal website [23], and normal tissue sample data from GTEx website. TCGA had characterized more than 20,000 primary cancer samples and corresponding non carcinoma samples from 33 types of cancers. RNA-sequencing datasets and along with the corresponding clinical annotations were obtained. Using the rma function in the R package (R version: 4.2.2), the whole data set was filtered, deleting missing and duplicated results, and transformed by log2(TPM + 1). To compare LFNG, MFNG and RFNG expression levels between the cancerous and normal samples, data regarding gene expressions were extracted from the 33 TCGA cancer types to form an expression matrix. Tumor stages, and clinical stages of patients were all retrieved from the portal websites, along with other clinical data.

### Cox regression analysis and survival analysis

We used Cox regression analysis to assess the significance of LFNG, MFNG, and RFNG in predicting Overall Survival (OS), disease-specific survival (DSS), disease-free interval (DFI), and progression-free interval (PFI) in pan-cancer using the using the log2-transformed TPM expression value of the three gene from TCGA database. OS was defined as the duration from the date of diagnosis to death from any cause. For DSS, patients who died from causes other than the specified disease were not included. PFI was defined as disease progression or death from any cause, while DFI included only patients who had disease progression. The Kaplan–Meier (KM) method was used to create survival curves for patients in each cancer type after separating them into high and low LFNG, MFNG, and RFNG expression subgroups with median value as thresholds (Supplement Table I). The survival analysis was performed using survival ROC and survival in the R package [24]. We used the log-rank test to compare differences between curves, with a *P*-value less than 0.05 considered significant. A survival-associated forest plot was then created, and we conducted KM analysis to compare OS for TCGA cancer patients stratified according to median LFNG, MFNG, and RFNG expression levels using the log-rank test. The PrognoScan database (http://dna00.bio.kyutech.ac.jp/PrognoScan/) were used for further survival analysis of LFNG, MFNG and RFNG in several GEO datasets with different cancer types.

### Immune cell infiltration

We utilized the ESTIMATE algorithm, which uses transcriptional profiles of cancer samples to infer the content of tumor cells, as well as infiltrating immune and stromal cells. The algorithm produces three scores using single sample Gene Set Enrichment Analysis (ssGSEA): the stromal score, which indicates stromal cells within tumor tissues; the immune score, which assesses immunocyte infiltration within the tumor tissues; and the estimate score, which can infer the purity of the tumor. We estimated both immune and stromal scores for tumor tissues using the ESTIMATE algorithm based on corresponding transcription data. We then calculated the correlations between these scores and LFNG, RFNG, and MFNG expressions.

For further analysis, we used CIBERSORT, a highly accurate metagene tool that precisely estimates 22 phenotypes of human immunocytes, to compute the association of LFNG, MFNG, and RFNG expression with each leukocyte phenotype in 33 cancer types. We also examined the associations of LFNG, MFNG, and RFNG levels with tumor-infiltrating immunocyte gene markers [25–27]. Correlation analysis was used to determine statistical significance and Spearman’s correlation coefficient. An expression heat map was then plotted for each gene pair within a specific type of cancer.

### Gene set enrichment analyses

Correlation analyses of LFNG, MFNG and RFNG with all genes was performed using TCGA data. Pearson’s correlation coefficients were calculated. Genes correlated with LFNG, MFNG and RFNG (*P* < 0.05) were selected for GSEA, for possible underlying mechanisms based on the ‘Molecular Signatures Database’ Bioconductor (http://bioconductor.org/) and R software (http://r-project.org/) were used to plot enrichment maps to visualize our results [28].

### Cell culture and transfection

Pancreatic cells (PANC-1, MIA PaCa-2, CFPAC-1, BxPC-3 obtained from ATCC) were cultured per ATCC recommendations. Overexpression or knockdown of LFNG in pancreatic cancer cells were through construction and transfection of overexpression vetocr pcDNA3.1-LFNG and shRNA-LFNG, respectively, and pcDNA3.1-NC and shRNA-NC as the corresponding control (Focus Bioscience Co., Ltd, Nanchang, China). One day before transfection, cells (1 × 10^6^/ml) were cultured in a 60-mm dish at approximately 50–60% confluence, and then transfection was carried out with Lipofectamine 3000 (Thermo Fisher Scientific, Inc.). Following 6 h of incubation, the old medium was discarded and fresh medium supplemented with 10% FBS was added.

### Quantitative RT‑PCR analysis

Total RNA was obtained using TRIzol reagent (Thermo Fisher Scientific, Inc.) according to the previous report, then 2 µg RNA was used to synthesize cDNA using the PrimeScript RTreagent kit (Takara, DRR037A). The amplification reaction was proceeded using SYBR-Green Advantage qPCR Premix (Takara) in the QuantStudio 5 Real-Time PCR‑System (Thermo Fisher Scientific, Inc.) with specific primers: LFNG forward primer: 5’-GACATTCAGGTAGAGACGTTCA-3’; LFNG reverse primer: 5’-ATACTCCACGGCCATCTTGC-3’. GADPH (F: 5’-GTCTCCTCTGACTTCAACAGCG-3’, R: 5’-ACCACCCTGTTGCTGTAGCCAA-3’) and U6 (F: 5’-CTCGCTTCGGCAGCACAT-3’, R: 5’-TTTGCGTGTCATCCTTGCG-3’) were used as endogenous controls, and relative expression level was computed using 2^−ΔΔCt^ method.

### Western blot detection

Cells were washed with cold PBS and lysed with WB cell lysis solution (Thermo Fisher Scientific, #89,900) and protease inhibitor cocktail (MCE, HY-K0010). Proteins were standardized using the BCA protein assay kit (Thermo Fisher Scientifc, #23,225). Then equal amounts of protein were subjected to SDS-PAGE and transferred to 0.45 μm PVDF membranes (Sigma Aldrich). The membranes were blocked with 5% defatted milk (1 h at room temperature), washed with TBST (PBS, 0.1% Tween 20), then incubated with primary antibody overnight at 4˚C. Washed 3 times with TBST, incubated with secondary antibodies (1:1000) at room temperature for 1 h and washed again with PBST. The primary antibodies were LFNG (1:1,000 dilution; CST, #66472S), GAPDH (1:10,000 dilution; Santa Cruz, #SC-47,724), and incubated overnight at 4 ˚C. Finally, the membranes were detected with ECL reagents (Biosharp, #BL520A) using Image Lab software.

### Invasion and migration assays

The transwell migration assay and transwell invasion assay were conducted with a Corning Inc. transwell chamber. Migration assay was used to determine the number of cells that traversed a porous polycarbonate membrane by using the 24‑well Transwell (corning #3422). In the assays, 1.5 × 10^5^ cells per well were seeded in an upper chamber in serum free media for the migration and invasion assay, respectively. The lower chamber was filled with media containing 10% FBS. After 24 h, cells passing through polycarbonate membrane were stained and counted according to the manufacturer’s instructions. For the invasion assay, the upper compartment was precoated with 100 µl of BD BioCoat Matrigel invasion chambers (BD Bioscience, #356,231). All other processes were the same as for the transwell migration assay.

### Cell cycle analysis

Cells were cultured for 24 h after transfection, then collected and fixed with pre‑chilled 75% ethanol in phosphate‑buffered saline (PBS) for 24 h at ‑20˚C, then centrifuged at 100 x g for 5 min and washed three times with PBS. Then cells were resuspended in 50 µg/ml propidium iodide (BD Biosciences). Cell cycle distribution was determined by flow cytometry (CytoFLEX, Beckman Coulter, USA) and the data analysis using the software FlowJo V10.

### Pseudopodia immunofluorescence staining

Prepare 1× Fluorescein Phalloidin working solution, according to the instruction manual (MCE, # HY-K0902), Wash cells 2–3 times with PBS. Fix the cells in 3.7% methanol-free formaldehyde solution in PBS for 10–30 min at room temperature. Wash the fixed cells 2–3 times in PBS. Add 100 µL/well (96-well plate) of Fluorescein Phalloidin working solution into the fixed cells, and stain the cells at room temperature for 20 to 90 min. Rinse cells gently with PBS 2–3 times to remove excess Fluorescein Phalloidin. Run fluorescence microscope at desired Ex/Em wavelengths (Fluorescein Phalloidin, Ex/Em = 497/516 nm).

### Immunohistochemical (IHC) staining

Immunohistochemical (IHC) staining assay was performed on pancreatic cancer tissue microarray slides (Cat#HPanA170Su04) that purchased from Outdo Biotech Co. (Shanghai, China). A total of 170 pancreatic cancer tissue samples with prognosis information were included. The tissue slide was incubated with Anti-LFNG (abcam, #ab235534) (dilution 1:200) antibody diluted with 5% BSA/PBS overnight at 4˚C and all procedures were conducted according to the manufacturer’s instructions, and slides were scanned by NanoZoomer (Japan) followed by scoring based on the portion of positive signal cells. Evaluation of IHC staining was conducted by a board-certifed pathologist who was blinded to the patients’ clinical data [29]. Since there is no consensus regarding the cutoff value of LFNG protein expression, we determined negative if less than 5% of tumor cell were stained. On the contrary, the expression was determined positive if ≥ 5% of tumor cells were IHC-stained positive. For those with positive LFNG expression, we categorized “LFNG+” if 5–24% of cells were positive, “LFNG++” if 25–75% of cells were positive, and “LFNG+++” if more than 75% of cells were positive after IHC staining.

### Drug response

We further applied pRRophetic package to estimate the correlation between the expression of LFNG and the drug response in PAAD and PRAD, which fitted the ridge regression model based on baseline gene expression and drug sensitivity of the cell line, thus allowing the prediction of the clinical chemotherapeutic response using only patients’ baseline gene expression data [30].

### Statistical analyses

Survival analysis was conducted using Kaplan-Meier curves, log-rank tests, and Cox proportional hazards regression models. Correlations were assessed using Spearman’s test. All statistical analyses were performed using R version 4.2.2. Experiments were conducted in triplicate and statistical results were reported as mean ± standard deviation (SD) using GraphPad Prism Software (version 9.3, CA, United States). Data are presented as mean ± standard deviation. Student’s t-test was used to compare differences between two groups. Statistical significance was determined as *P*-value < 0.05.

## Results

### LFNG, MFNG and RFNG expressions analysis in pan-cancer

**Fig. 1:**
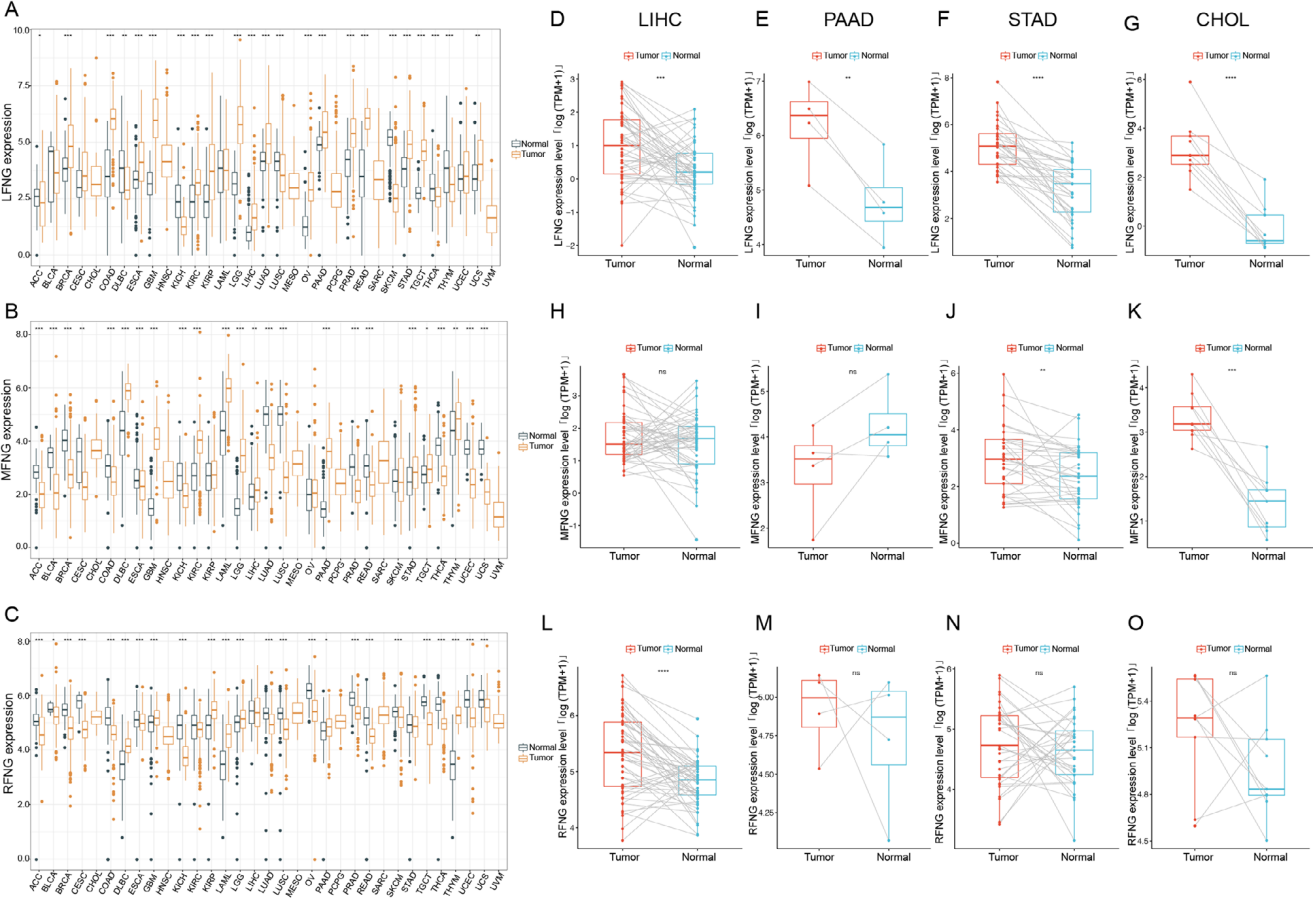
The differences expressions of LFNG, MFNG and RFNG in human tumors and healthy tissues among the 33 type cancers. (**A-C**). The black and yellow bar graphs indicate normal and tumour tissues of LFNG (**A**), MFNG (**B**) and RFNG (**C**), respectively. **D-O.** Pan-cancer differential expression of LFNG, MFNG and RFNG in paired tumor and adjacent normal tissues in indicated representative tumor types (LIHC, PAAD, STAD, CHOL) from TCGA database. The blue and red bar graphs indicate normal and tumor tissues of LFNG (**D-G**), MFNG (**H-K**), RFNG (L-O), respectively. (* represents *P* < 0.05, ** represents *P* < 0.01, *** represents *P* < 0.001)

To investigate the expression profiles of LFNG, MFNG, and RFNG in pan-cancer, we analyzed the expression status of these three genes across various cancer types in TCGA data. Our results showed that higher expressions of LFNG, MFNG, and RFNG were observed in 9 tumors compaied with their counterpart normal tissues, including glioblastoma multiforme(GBM), kidney chromophobe (KIRC), kidney renal papillary cell carcinoma (KIRP), acute myeloid leukemia (LAML), brain lower-grade glioma (LGG), liver hepatocellular carcinoma (LIHC), ovarian serous cystadenocarcinoma (OV), pancreatic adenocarcinoma (PAAD), and stomach adenocarcinoma (STAD) (Fig. 1A-C). We further evaluated the expression difference of LFNG, MFNG, and RFNG in paired between the normal tissues and tumor tissues in LIHC, PAAD, STAD and cholangiocarcinoma (CHOL) (Fig. 1D-O). The results were showed a significantly (P < 0.001) higher expression of LFNG in the tumor tissues than in normal tissues. The expression of MFNG in tumor tissue of STAD and CHOL and the expression of RFNG in tumor tissue of LIHC were also significantly (P < 0.001) higher than in normal tissue. Additionally, we evaluated the expression of LFNG, MFNG, and RFNG in different cancer stages. Our findings indicated that LFNG was highly expressed in higher stages of several cancers, including PAAD, LIHC, CHOL, and STAD, as well as in lung adenocarcinoma (LUAD), rectum adenocarcinoma (READ), esophageal carcinoma (ESCA), colon adenocarcinoma (COAD), breast invasive carcinoma (BRCA), kidney renal clear cell carcinoma (KIRC), testicular germ cell tumors (TGCT) and KIRP (Supplement Fig. 1). Overall, the same trend was observed in gastrointestinal system tumors, such as LIHC, PAAD, STAD and CHOL, the expressions of LFNG, MFNG and RFNG were generally at higher levels in theses tumors than in normal tissues (Fig. 1).

### CNV characteristics of LFNG, MFNG and RFNG

**Fig. 2:**
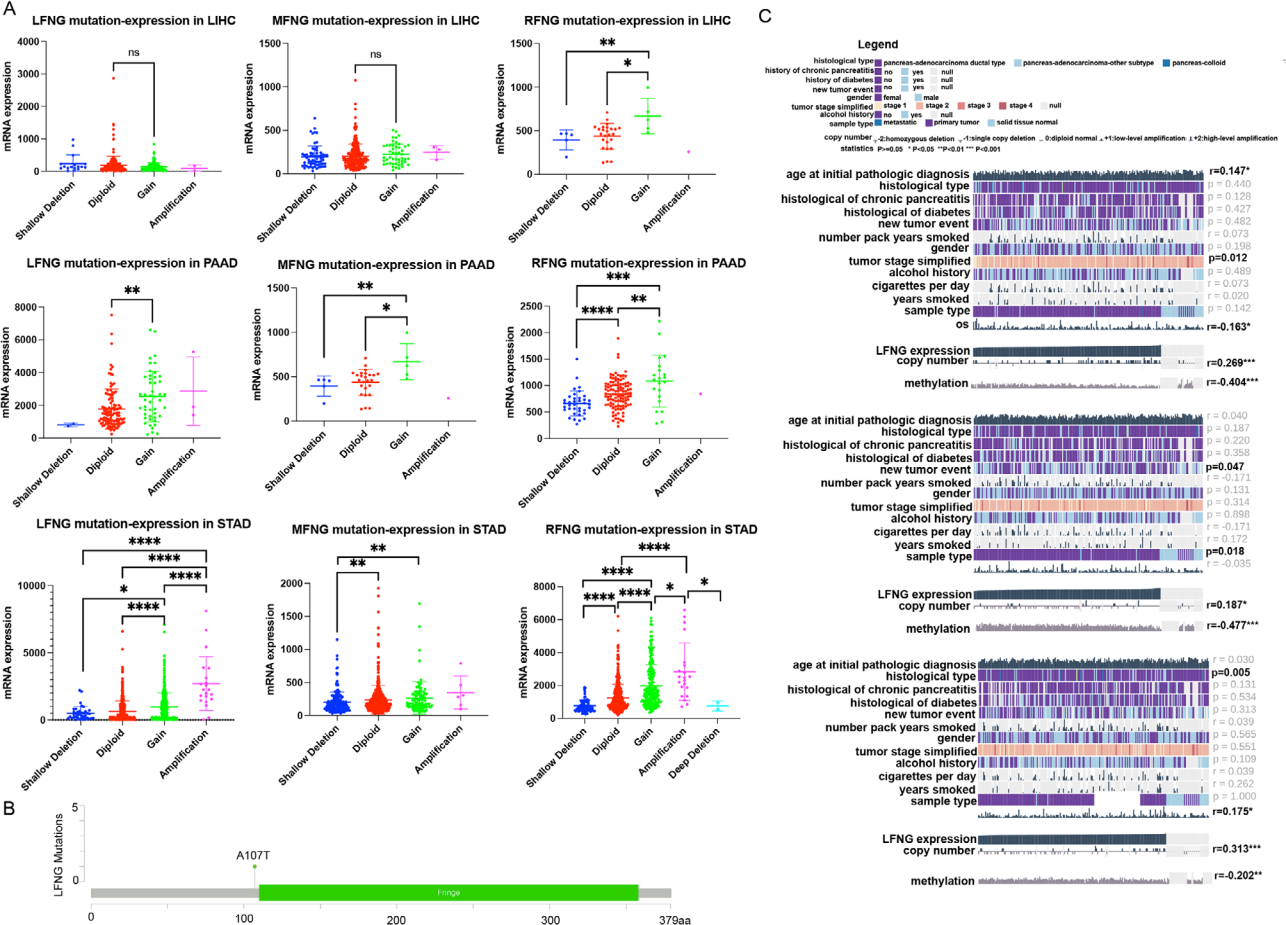
Mutation analysis of LFNG, MFNG and RFNG. (**A**) CNV analysis of LFNG, MFNG and RFNG in LIHC, PAAD and STAD, and the diploid, gain and amplification mutation were significantly increased compare with the shallow deletion. (**B**) Mutation site of LFNG is A107T which analyzed by cbioportal analysis. (**C**) The expression mutation of LFNG, MFNG and RFNG were significantly correlated with methylation and OS in PAAD patients based on MEXPRESS database (* represents *P* < 0.05, ** represents *P* < 0.01, *** represents *P* < 0.001)

Given the similar expression pattern of the Finger family in gastrointestinal system tumors, we investigated the relationship between mutation and expression of LFNG, MFNG, and RFNG in LIHC, PAAD, and STAD. As somatic CNV is highly associated with the prognosis of numerous cancers by impacting gene expression level. Given the similar expression pattern of the Finger family in gastrointestinal system tumors, we investigated the relationship between mutation and expression of LFNG, MFNG, and RFNG in LIHC, PAAD, and STAD. The gain and amlplification of LFNG, MFNG and RFNG were increased compare with their shallow deletion and diploid in LIHC, PAAD and STAD, especially LFNG amplification in PAAD and STAD (Fig. 2A). The LFNG mutation site was identified as A107T (Fig. 2B). An epigenetic mechanism well known as DNA methylation also influence transcript regulation, involved in cancer progression. Therefore, between the CNV and associated methylation characteristics and the expression of LFNG, MFNG and RFNG was analyzed. We found that the LFNG, MFNG and RFNG expression were negatively associated with their DNA methylation status, and further confirmed their expression positively correlated with CNV. Our results further revealed a significant correlation between copy number variation, methylation, and overall survival, which was showed that methylation of LFNG and MFNG was negatively correlated with prognosis and was significant, which was contrary to RFNG. And the CNV of these genes were positively associated with prognosis (Fig. 2C). Our results further revealed a significant correlation between copy number variation, methylation, and overall survival, particularly in the LFNG and RFNG genes.

### Analysis of relationship between LFNG, MFNG and RFNG expression levels and prognosis in pan-cancer

**Fig. 3:**
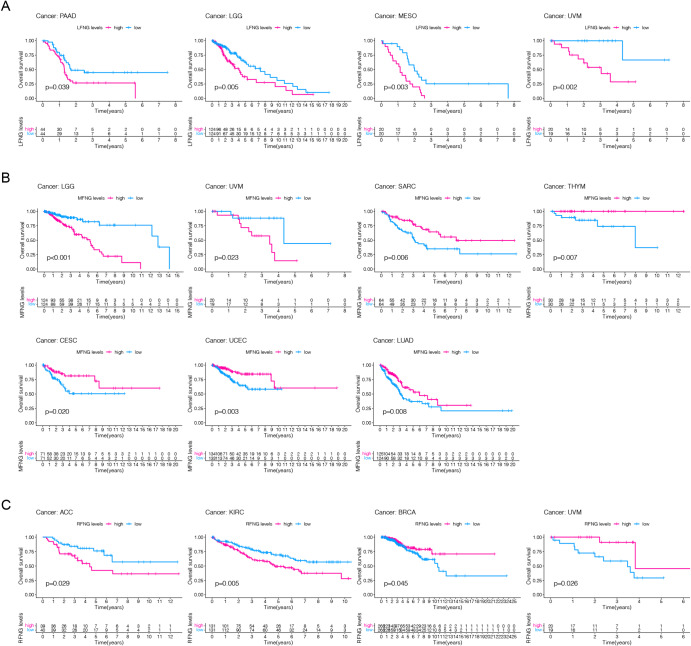
Correlation between survival and LFNG, MFNG, RFNG gene expression in different cancer types. **A–C.** Kaplan-Meier overall survival of LFNG (**A**), MFNG(**B**) and RFNG(**C**) in indicated representative tumor types. The median value of LFNG, MFNG and RFNG in each tumor was taken as the cut-off value. (* represents *P* < 0.05, ** represents *P* < 0.01, *** represents *P* < 0.001)

We conducted further analysis to assess the prognostic significance of LFNG, MFNG, and RFNG expression in different cancer types. Our results showed that high expression of LFNG was associated with poor prognosis in patients with mesothelioma (MESO), uveal melanoma (UVM), LGG, and PAAD (Fig. 3A). High expression of MFNG was also indicative of poor prognosis in patients with LUAD, thymoma (THYM), uterine corpus endometrial carcinoma (UCEC), endocervical adenocarcinoma (CESC), (sarcoma) SARC, and THYM (Fig. 3B). On the other hand, high expression of RFNG was significantly associated with poor prognosis in patients with adrenocortical carcinoma (ACC) and KIRC. Interestingly, low expression of RFNG was also found to predict poor prognosis in some tumors, such as UVM and BRCA (Fig. 3C).

**Fig. 4:**
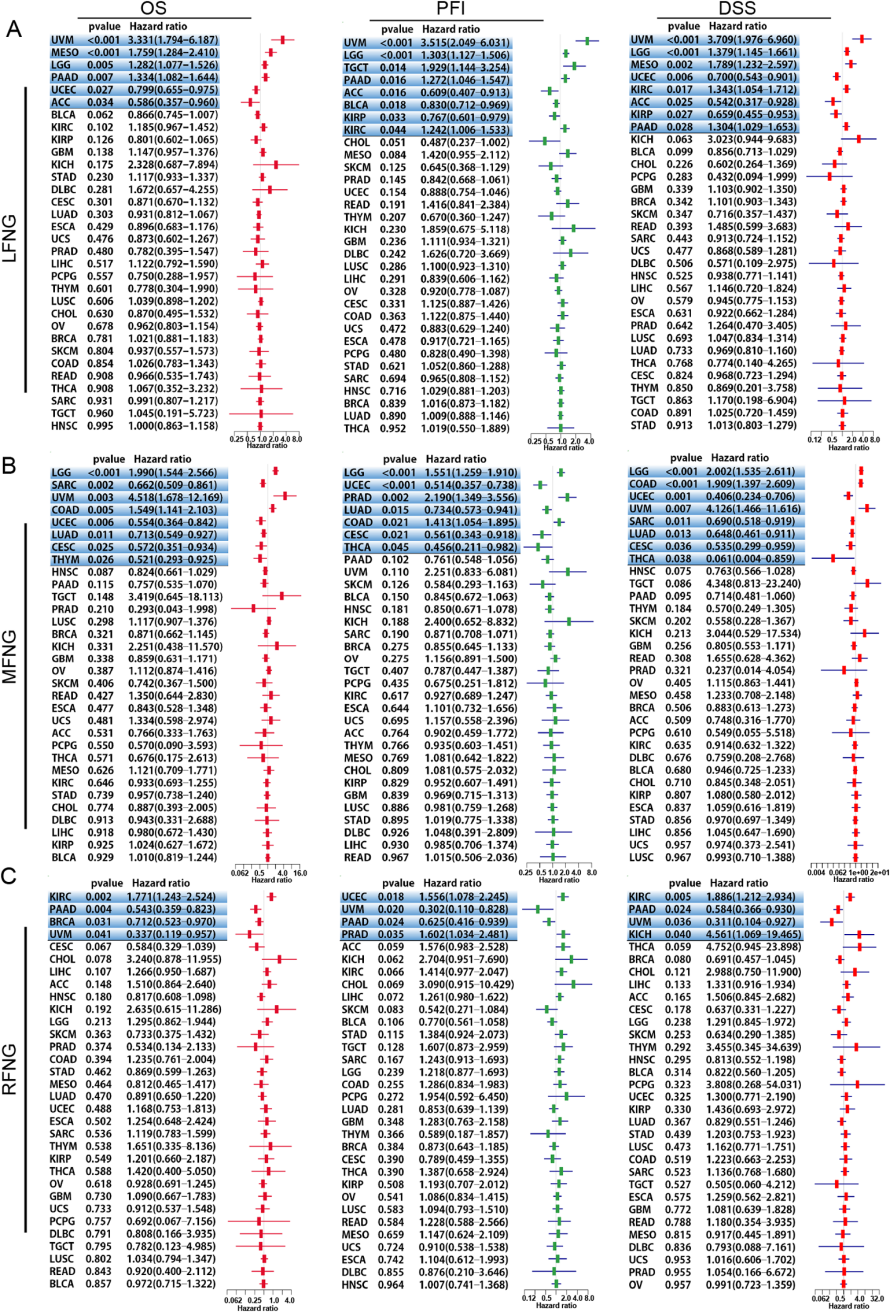
Univariate Cox regression analysis of LFNG, MFNG and RFNG. (**A-C**). The forest plot shows the relationship of LFNG (**A**), MFNG (**B**) and RFNG (**C**) expression with patient overall survival (OS), disease-specific survival (DSS), disease-free interval (DFI) and progression-free interval (PFI). (* represents *P* < 0.05, ** represents *P* < 0.01, *** represents *P* < 0.001)

We performed single variate Cox regression analysis to examine the relationship between LFNG, MFNG, and RFNG expression levels and overall survival (OS), disease-specific survival (DSS), disease-free interval (DFI), and progression-free interval (PFI) in different cancer types. High expression of LFNG was found to be a risk factor for OS, DSS, and PFI in most cancer types, except for UCEC, KIRP, and ACC, where it acted as a protective factor (Fig. 4A). For MFNG, high expression was a risk factor for OS, DSS, and PFI in LGG, COAD, and UVM, but a protective factor for SARC, UCEC, LUAD, CESC, and THYM (Fig. 4B). High expression of RFNG was found to be a risk factor for KIRC, DSS, and PFI, while it was a protective factor for PAAD, BRCA, and UVM (Fig. 4C). The data of DFI was shown in Supplement Fig. 2. The prognostic capabilities of these three genes was also verified through PrognoScan data sets (PrognoScan_result.xlsx) as Supplement Table II. This result showed that the expression of LFNG, MFNG and RFNG were significantly associated with the prognosis of blood cancer, brain cancer, breast cancer, colorectal cancer, lung cancer.In addition, a verification of the prognostic discrimination of LFNG in pancreatic cancer by means of pancreatic cancer data set GSE28735 (Supplement Table III) was shown in Supplement Fig. 3 (Survival proportions of GSE28735) .

Taken together, our findings suggest that LFNG and MFNG are common risk factors in PAAD, UVM, and LGG, and their high expression levels indicate poor prognosis. High expression of RFNG is also a risk factor in some cancer types, while low expression can also predict poor prognosis in certain tumors. These results highlight the importance of LFNG, MFNG, and RFNG as potential prognostic markers and therapeutic targets in cancer.

### Correlation between LFNG, MFNG and RFNG expression and immune infiltrating level in cancers

**Fig. 5:**
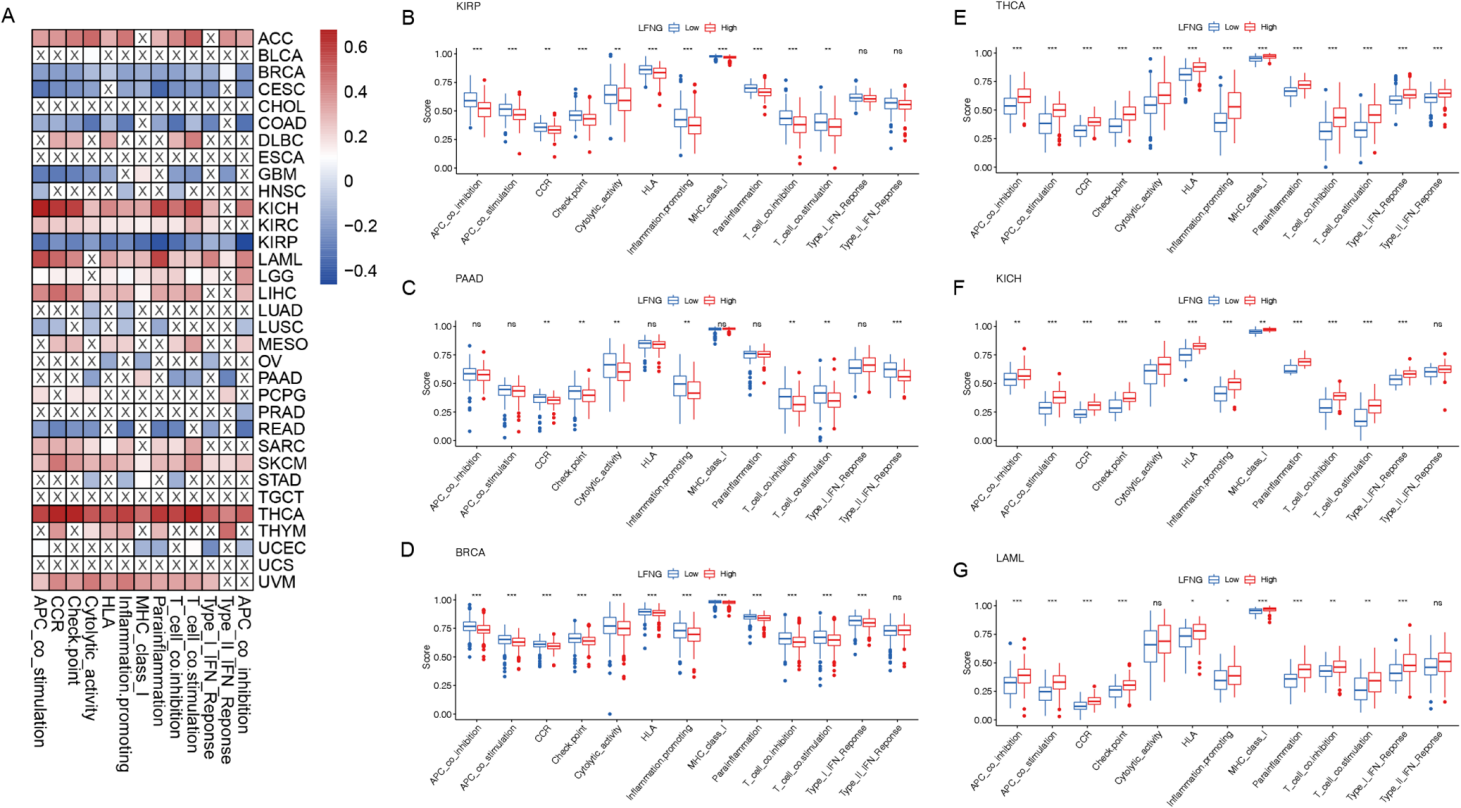
The effect of LFNG on immunological status in pan-cancer. (**A**). Correlation between LFNG and 13 tumor-associated immune cells, as calculated using the ssGSEA algorithm. (**B-G).** Differences in the various immune cells proportion between groups with high and low LFNG expression in representative cancers. (ns, no significant difference. *P < 0.05; **P < 0.01; ***P < 0.001; ****P < 0.0001)

Cytokines and their surface receptors are glycoproteins, and the expression levels of LFNG, MFNG, and RFNG, as three key genes in the process of glycosylation regulation, can affect cytokine secretion and thus regulate tumor immune infiltration. Therefore, we further analyzed the relationship between their expressions and immune cell infiltration. Most immune cells were negatively correlated with the expression of LFNG, MFNG, and RFNG in pan-cancers, especially T cells (Supplementary Fig. 4, Supplementary Fig. 5, Supplementary Fig. 6). As LFNG has better activity compared to MFNG and RFNG and has a more significant influence on prognosis in cancers, we showed the immune cell infiltration and single immune cell type corresponding to the expression of LFNG in Fig. 5. High expression of LFNG was significantly associated with a low score of immune cells in BRCA, CESC, COAD, GBM, KIRP, PAAD, and READ (Fig. 5B-D). However, there was a significantly positive association in ACC, CHOL, KICH, KIRC, LAML, LIHC, SARC, SKCM, and THCA (Fig. 5E-G).

### Enrichment analysis of LFNG, MFNG and RFNG in cancers

To assess the biological effects of LFNG, MFNG, and RFNG expression, we performed GSEA using multiomic data from TCGA. We found that the main GO enrichment pathways in the high expression group of LFNG, MFNG, and RFNG in pan-cancer were similar and could be classified into three categories: immune response and T cell activation pathways, extracellular matrix adhesion pathways, and RNA splicing and ion transport pathways. The representative cancer types are shown in Supplement Fig. 7. In cancers such as CHOL, BRCA, COAD, GBM, KIRC, LAML, LGG, LIHC, TGCT, LUAD, and PAAD, the immune pathway is the main pathway enriched (Supplement Fig. 7A-C). Cancers represented by collagen-containing extracellular matrix/cell substrate adhesion and Jak-STAT signaling pathway include KICH, OV, PRAD, and STAD. Notably, the expressions of LFNG, MFNG, and RFNG in gastrointestinal tumors LIHC, STAD, and PAAD were all enriched in immune response and intercellular adhesion pathways. These results suggest that LFNG, MFNG, and RFNG may promote tumor progression through immune and intercellular regulatory effects.

### LFNG promote Pancreatic cancer cell proliferation, invasion and cell cycle

**Fig. 6:**
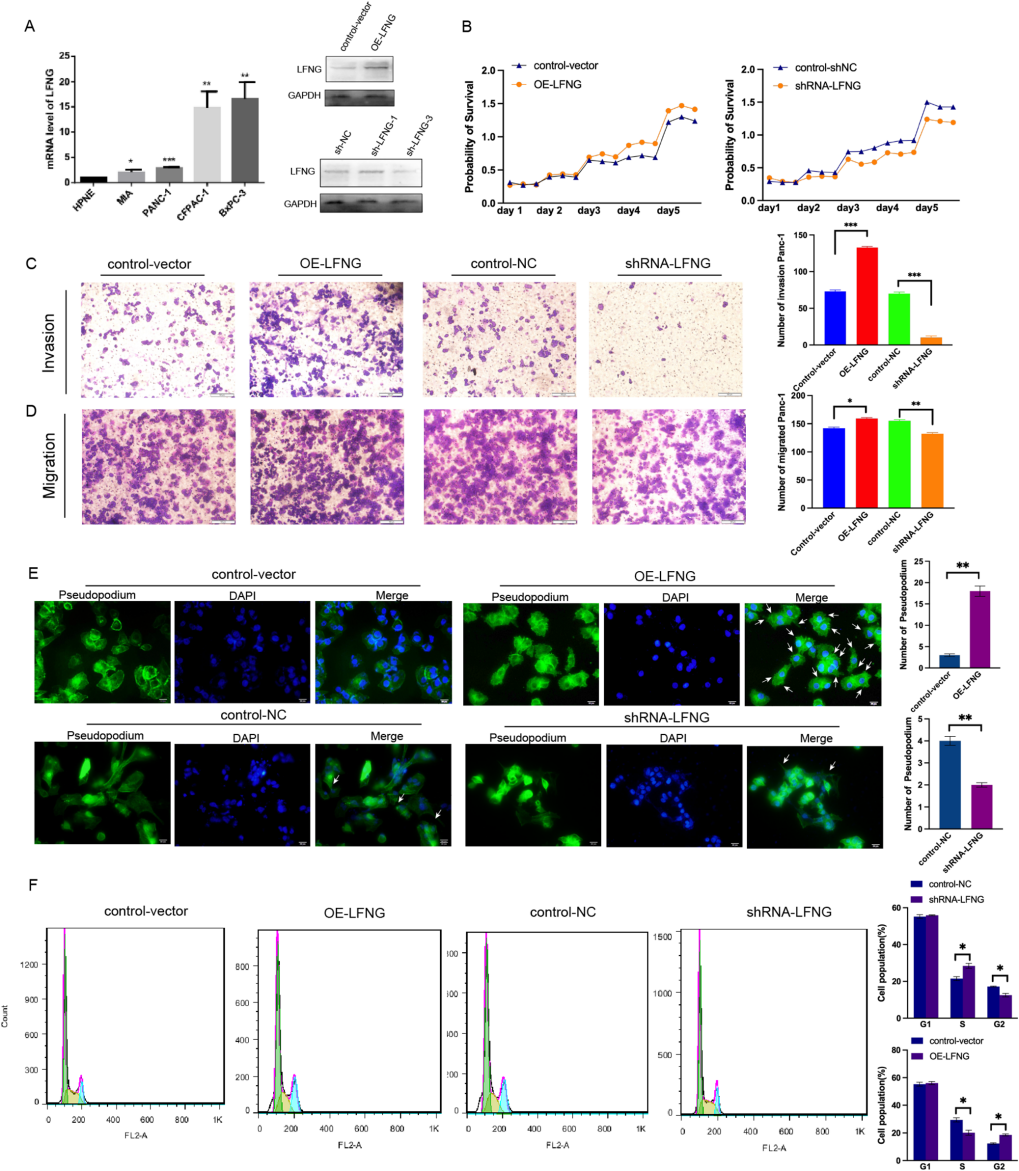
The effects of LFNG on proliferation, migration, invasion and cell cycle of PC cells. (**A**) The expression levels of LFNG in different pancreatic cancer cell lines. LFNG-overexpressed and shRNA-LFNG and the control vector or mimic (NC) were transfected into PANC-1 cells. Relative expression of LFNG was determined 24 h after transfection by western blot. (**B**) Overexpressed of LFNG observably promoted the proliferation ability in PC cells compared with the NC group. **C.D** Overexpressed of LFNG could significantly increase the migration and invasion of PC cells. **E.** The number of pseudopodium formation was significantly increased under LFNG overexpressed compare with the control cells. **F.** LFNG regulates cell cycle progression in PANC-1 cells. Cell cycle distribution was analyzed 24 h after transfection using flow cytometry. Values are presented as the mean ± standard deviation (n ≥ 3) (*P < 0.05, **P < 0.01)

After analyzing the expression levels of LFNG, MFNG and RFNG in various cancer types, the present study focused on LFNG in pancreatic cancer (PAAD), where its abnormal expression was found to be associated with poor prognosis [31]. To investigate its function in PCa cells, PANC-1 cells were transfected with an overexpression vector (pcDNA3.1-LFNG) or a knockdown RNA (shRNA-LFNG). Western blotting confirmed the successful modulation of LFNG expression (Fig. 6A). In our experiment, we made three shRNAs of LFNG to knockdown LFNG expression, the the result suggested a more effectively function of shRNA3 in knockdown LFNG as shown in Fig. 6, then shRNA3 was used in further study. Overexpression of LFNG significantly increased cell survival (Fig. 6B) and promoted invasion and migration of PANC-1 cells (Fig. 6C-D). This result was further confirmed by pseudopodia experiments, which revealed a significant increase in pseudopodia number under overexpressed LFNG (Fig. 6E). Furthermore, flow cytometry analysis showed that LFNG overexpression resulted in G2 phase arrest, while knockdown of LFNG increased the proportion of cells in S phase, but decreased G1 and G2 phase cells (Fig. 6F). These findings suggest that LFNG may regulate cell cycle progression and promote tumor invasion, possibly by promoting cell proliferation and suppressing apoptosis.

### High expression of LFNG in Pancreatic cancer patients indicated a poor prognosis

**Fig. 7:**
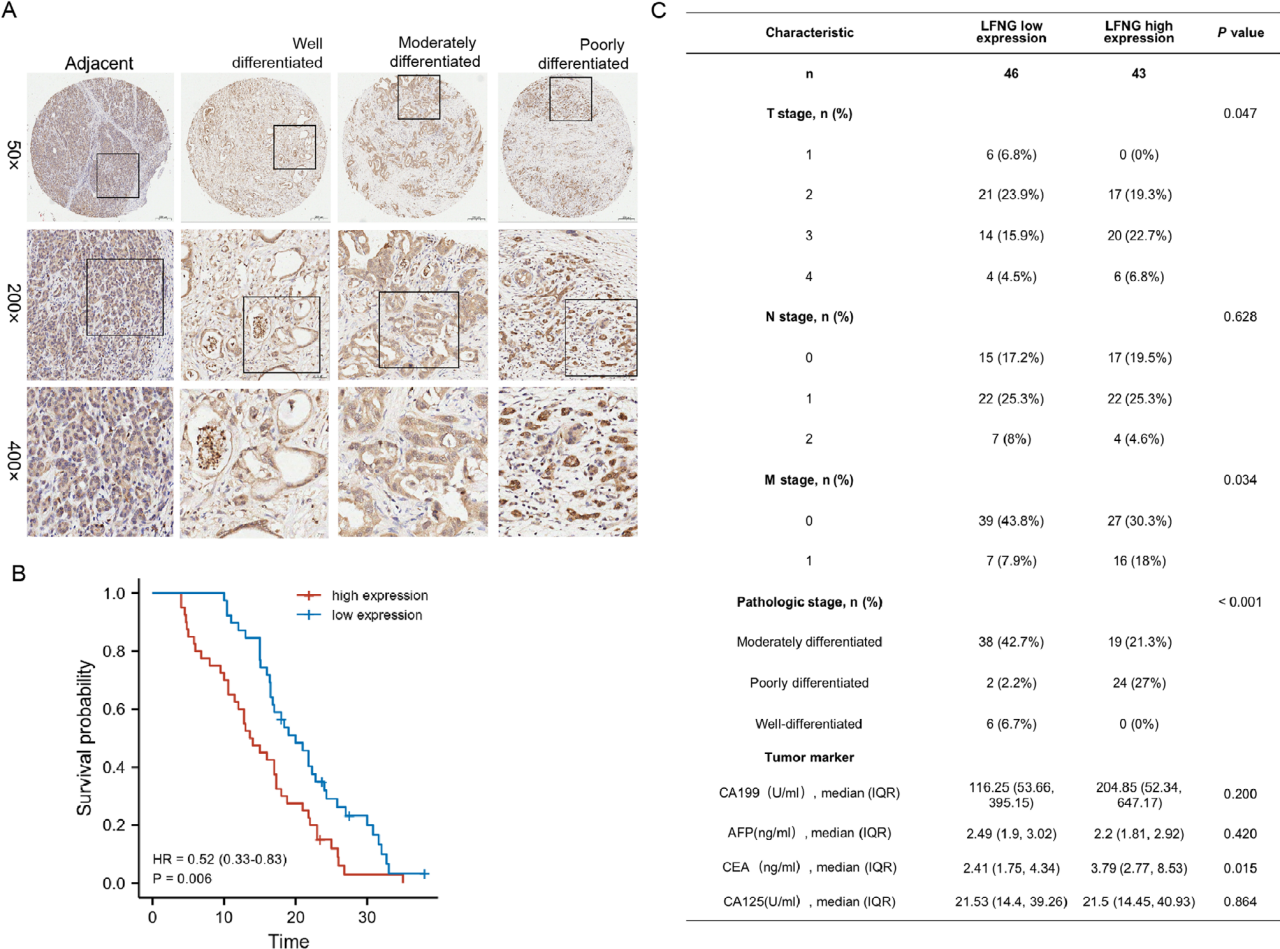
Validation of the prognosis predictive ability of LFNG in a clinical external cohort. (**A**) Representative images of IHC (50×, 200×, 400× magnification) of LFNG in PC patients tissue samples. (**B**) The correlation between overall survival based on clinical information and the expression of LFNG in PC patients tissue samples. Compared with the low expression, the high expression of LFNG has a significantly lower overall survival rate. (**C**) Clinicopathologic data showed that LFNG expression was significantly correlated with tumor T stage, differentiation degree, pathological grade and distant metastasis of pancreatic cancer. (log-rank test, *P < 0.05)

A clinical cohort comprising of 90 pancreatic cancer (PC) patients at different clinical stages was established to validate the predictive ability of LFNG expression. IHC analysis showed that LFNG expressions varied significantly across different stages of PC. LFNG expressions were scored for each pancreatic cancer patient based on the positive expression of LFNG in the tissue sample (e.g., 25% positive expression rate = 1 point, 50% positive expression rate = 2 points, 75% positive expression rate = 3 points, 100 positive expression rate = 4 points). Subsequently, we calculated the risk score based on LFNG expressions and found that high LFNG expression was associated with poor survival prognosis (*P* < 0.05) in pancreatic cancer patients (Fig. 7). LFNG expression levels were found to increase gradually in paracancer tissues, and were high in moderately to poorly differentiated tumors (Fig. 7A). Compared to low LFNG expression, high LFNG expression was significantly associated with a lower overall survival rate (Fig. 7B). The clinicopathological analysis revealed that LFNG expression was significantly correlated with tumor T stage, differentiation degree, pathological grade, and distant metastasis in pancreatic cancer patients (log-rank test, *P < 0.05) (Fig. 7C). These results suggest that high LFNG expression level in pancreatic cancer patients was predictive of poor prognosis and are positively associated with poorly differentiated tumors and distant metastasis.

### Analysis of drug sensitivity between the high-expression and the low-expression LFNG in PAAD and PRAD

**Fig. 8:**
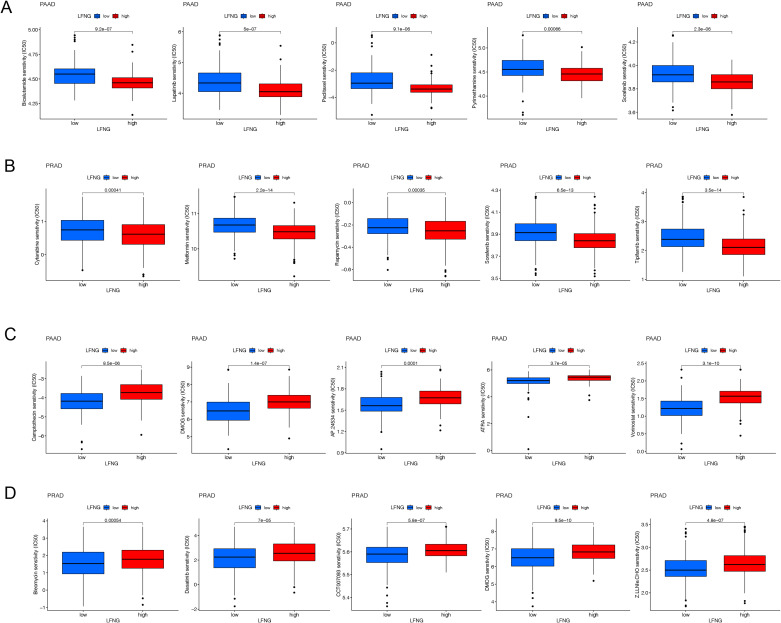
Analysis of drug sensitivity based on the LFNG expression in PAAD and PRAD. (**A**, **B**) Bicalutamide, lapatinib, Paclitaxel, pyrimethamine, Sorafenib, and Cytarabine, Metformin, Rapamycin, Sorafenib, Tipifarnib were relatively sensitive in PAAD and PRAD patients with higher expression of LFNG (*P < 0.05). (**C**, **D**) Camptothecin, DMOG, AP.24,534, ATRA, Vorinostat and Bleomycin, Dasatinib, CCT007093, DMOG, Z.LLNle.CHO were relatively resistant in LFNG higher expression PAAD and PRAD. (*P < 0.05)

Furthermore, we assessed the sensitivity of potential drugs correlated with LFNG expression levels in PAAD and PRAD by the pRRophetic package. In PAAD and PRAD with high expression of LFNG group, there was a more sensitive to Bicalutamide, lapatinib, Paclitaxel, pyrimethamine, Sorafenib and Cytarabine, Metformin, Rapamycin, Sorafenib, Tipifarnib, respectively (*P* < 0.001) (Fig. 8A-B). However, there was a relatively resistant to Camptothecin, DMOG, AP.24,534, ATRA, Vorinostat and Bleomycin, Dasatinib, CCT007093, DMOG, Z.LLNle.CHO in higher LFNG expression PAAD and PRAD (Fig. 8C-D). This analyses suggested that PAAD patients with high expression of LFNG gene may have a higher probability of benefiting from a combination treatment of paclitaxel and sorafenib. However, this hypothesis needs to be validated in future clinical trials.

## Discussion

Although Fringe family was initially reported to be involved in embryonic development, many recent studies have shown that they play a significant role in various cancers due to the significant changes in the glycosylation modification of cells in malignant tumors [32, 33]. Among the human Fringes, LFNG, MFNG, and RFNG are relatively understudied, despite their biological importance [34]. A pan-cancer analysis of LFNG, MFNG, and RFNG is therefore necessary. Previous studies have revealed the role of Fringe genes in the antagonistic and agonistic functions of Notch toward its ligands, and the different expression patterns of Fringe genes during carcinogenesis [35].

Our study showed that LFNG was generally highly expressed in tumors compared to MFNG and RFNG. Interestingly, we found that in the analysis of pathological stages, there was an increased expression of LFNG in higher stages of patients, while the expressions of MFNG and RFNG were decreased. This suggests that the expression patterns of LFNG, MFNG, and RFNG differ in different tumors. We focused on gastrointestinal cancer, and the copy number variation (CNV) of LFNG, MFNG, and RFNG were significantly changed in LIHC, STAD, and PAAD, consistent with the unusual glycosylation significantly affecting the digestive system. In our study, LFNG, MFNG and RFNG expression were negatively associated with their DNA methylation status, this was consistent with a LFNG and its DNA methylation in breast cancer. As we known, DNA methylation play a crucial role in mediating gene expression. In this study, LFNG is expressed at a high level in tumors, and its high level of transcriptional activity leads to its stronger regulatory effect on downstream NOTCH, which may lead to stronger invasion and migration ability of tumor cells. Multiple studies have reported different results regarding the associations with variation characteristics of methylation status, subtypes, and tumor grade/stage status of Notch-related components expressions [36]. This was first study suggested there were a negative correlation between LFNG, MFNG and RFNG expression and its DNA methylation, suggesting a critical role of epigenetic regulation, one potential mechanism is the activation of NOTCH signaling by LFNG, MFNG and RFNG, resulting in invasive growth and migration of pancreatic cancer cells. Furthermore, we found that LFNG, MFNG, and RFNG were significantly associated with the prognosis of pan-cancers by KM and COX analysis. Our results showed that the effects of LFNG and MFNG in PAAD, UVM, and LGG were risk factors, while RFNG in PAAD and UVM was a protective factor. Fringe family plays an important role in NOTCH signal transduction pathway. The mechanism of LFNG, MFNG, RFNG genes affecting the survival rate of cancer patients may be as follows: firstly, promote tumor development and metastasis: the expression levels of LFNG, MFNG and RFNG genes may lead to abnormal activation/inactivation of Notch pathway, thus promoting the proliferation, invasion and metastasis of tumor cells, thereby affecting the survival rate of cancer patients [22]. Further, the MFNG gene plays a role in regulating cell adhesion and migration. The study found that the deletion or reduced expression level of MFNG gene may lead to changes in cell polarity, which in turn increases the invasion and metastasis ability of cancer cells, thereby affecting the survival rate of patients [37]. Secondly, influence tumor immune response: LFNG, MFNG, RFNG gene expression levels may regulate the activation and function of immune cells [11]. Mutations in the LFNG gene may lead to an impaired immune response, thereby reducing the survival rate of patients. Recent studies have found that mutations in the LFNG gene can cause tumor cells to reduce their ability to recognize by the immune cells, thereby evading detection by the immune system [38]. Thirdly, LFNG gene mutation may be related to tumor cell resistance to drug therapy [39]. Some studies have found that mutations in the LFNG gene are associated with resistance of tumor cells to chemotherapy drugs, which can lead to a diminished response to treatment and thus affect survival [39]. At the same time, RFNG gene is involved in the degradation, transport and repair processes of tumor cells to chemotherapy drugs, and mutation or deletion may lead to the abnormality of these processes, making tumor cells resistant to chemotherapy drugs [40]. In conclusion, the expression of LFNG, MFNG, and RFNG may affect survival of cancer patients through a variety of mechanisms, including promoting tumor development and progression, affecting tumor immune response, and drug resistance. However, the specific mechanism still needs further research to clarify.

Abnormal tumor glycosylation alters the way the immune system perceives the tumor, inducing immunosuppressive signals [41]. Therefore, the specific glycan signal found on tumor cells can be considered as a new type of immune checkpoint [42]. In recent years, glycosylation has become a new hallmark of cancer [43, 44]. We analyzed the relationship between the expression of LFNG, MFNG, and RFNG and immune cell infiltration among the 33 types of cancers in this study. There was a higher expression of LFNG, MFNG, and RFNG associated with a lower proportion of immune cells in most cancers, suggesting an immune suppression microenvironment in Fringe higher expressed cancers. We also provided evidence processed by GO analysis, and the most correlated pathways of LFNG, MFNG, and RFNG in pan-cancer were in the immune response pathway, extracellular matrix adhesion pathway, and RNA splicing ion transport pathway, indicating that immune regulation was an essential pathway regulated by LFNG, MFNG, and RFNG. This may be a new perspective to explore the immune mechanism and immunotherapy for cancer.Notably, studies have reported Notch signaling has been implied in mediating chemotaxis system CXCL12/CXCR4 promoted carcinoma metastasis and multiple myelom [45]. The expressions of CXCR4 and CXCL12 were detected through qRT-PCR and western blot in PAAD due to LFNG dominant role in CNV expression, prognostic indicator, and functional enrichment in this study. The results shown a significantly down expression of CXCR4 in shRNA-LFNG transfected pancreatic cancer cells, and increased expression in LFNG over-expression pancreatic cancer cells, indicated LFNG promoted pancreatic cancer progression probably related to the increased expression of CXCR4 expression.

Previous studies have shown that LFNG decrease could be used as a biomarker in basal-like mammary tumors [46], while MFNG functions as an oncogene in breast cancer and contributes to the aggressiveness of CLBC [19]. Moreover, LFNG knockdown in the mouse prostate gland resulted in differential Notch regulation [13], suggesting that the Fringes could modulate distinct epithelial cell types. In contrast, the opposing dual roles of LFNG in PDAC, a very aggressive malignancy that usually defies most therapies, require further investigation. Our study aimed to verify the functional role of LFNG in PAAD, a type of pancreatic cancer, through in vitro experiments. We found that overexpressing LFNG significantly increased proliferation, migration, and invasion of pancreatic cells, as evidenced by pseudopodia analysis. This suggested that up-regulated LFNG expression was associated with increased invasiveness in PAAD. Glycosylated modified proteins are closely related to the cell cycle [47]. In our study, knockdown of LFNG led to an increase in the S phase without an equally significant decrease in G0/G1, which should indicate cell inhibition. This suggests that tasks that should be accomplished in the S phase are not completed, or there is a problem with the checkpoint. Both the S phase and G2 phase are periods of DNA replication, and the G2 phase is a period of high RNA and protein synthesis, especially tubulin [48]. Therefore, overexpression of LFNG promotes cell proliferation and pseudopodia formation. A prior study has revealed the expression of more than one Fringe within the same cell type. For instance, LFNG, MFNG, and RFNG are expressed in T cell progenitors and that the expression of all three Fringes is needed for optimal NOTCH1-dependent T cell development [49]. Nonetheless, the expression of LFNG alone (double knockout of Mfng and Rfng) resulted in similar levels of thymocytes to that in WT mice, while the presence of MFNG or RFNG alone resulted in thymocyte levels similar to triple-Fringe KO mice, suggest that while all Fringes are needed for optimal T cell development, LFNG provides a higher level of NOTCH1 activation [50]. LFNG had a dominant effect over MFNG or RFNG on NOTCH1 activation by DLL1 in cell-based NOTCH1 signaling assays. This dominance was due to the ability of LFNG to more extensively modify O-fucose residues present on EGF8 and 12, both of which play a key role in the activation of NOTCH1 by DLL1 mass spectral glycoproteomic analysis. Glycoproteomic analysis showed that co-expression of either LFNG or MFNG with RFNG resulted in modification of EGF6, reducing activation of NOTCH1 by JAG1 [51]. Totally, study have suggested that there is no competition or interaction between LFNG, MFNG, and RFNG, but, the combination of multiple Fringes leads to a level of elongation similar to the elongation observed for the most efficient Fringe-LFNG. These results show a hierarchy of Fringe activity and indicate that the effect of MFNG and/or RFNG could be small in the presence of LFNG. In this paper, we only analyzed the impact of each member on cancer patients, and there was no data on the impact of co-expression on prognosis. However, based on previous literature reports and findings in this study, compared with MFNG and RFNG, LFNG is still a more important member for cancer prognosis. This is also the reason why the pancreatic cancer cohort chip was used for verification in this study. We found that patients with poorly differentiated pancreatic cancer had higher LFNG expression and worse prognosis, suggesting LFNG as a candidate prognosis predictor in PAAD. The potential mechanism maybe the specific activity of LFNG for modifying O-fucosylated NOTCH1 EGF26 is 6 times higher than RFNG and 150 times higher than MFNG. Consequently, the observed dominance of LFNG over MFNG and RFNG could be correlated to the higher enzymatic activity. Therefore, modulated the enzymatic activity of LFNG might a way to improve prognosis of pancreatic cancer. Drug resistance is a critical factor in poor prognosis, particularly in PAAD and PRAD. Hyperactivation of specific signaling pathways, including Notch signaling, may enable cancer cells to evade the cell death-inducing effect of chemotherapy. We performed a drug-sensitive screen through CGP to find a suitable therapeutic strategy for LFNG-expressed PAAD and PRAD. Overall, mechanistic studies on Fringes could provide novel therapeutic and diagnostic approaches for cancer therapy.

In conclusion, our pan-cancer analyses have revealed correlations between LFNG, MFNG, and RFNG expressions, with its methylation states, mutations, clinical prognosis, and immune cell infiltration. These findings provide a better understanding of the role of the Fringe family in tumorigenesis. In future studies, it would be important to explore in-depth the regulation mechanisms between LFNG, MFNG, and RFNG methylation and expression and antitumor immunity. Based on our analysis, we have confirmed and discussed LFNG as a new potential therapeutic target for pancreatic cancer. LFNG may also be a valuable predictive biomarker for clinical outcome in multiple cancer therapies. Overall, our findings have important implications for the development of new therapeutic strategies and biomarkers for cancer treatment.

### Electronic supplementary material

Below is the link to the electronic supplementary material.

Supplementary Material 1

## Data Availability

The datasets generated and/or analyzed during the current study are available in the TCGA and UCSC Xena repository, https://www.cancer.gov/ccg/research/genome-sequencing/tcga, https://xena.ucsc.edu.
