# Supplementary material for: A pan-cancer analysis revealing the role of LFNG, MFNG and RFNG in tumor prognosis and microenvironment
Source: BMC Cancer. 2023 Nov 6;23:1065. doi: 10.1186/s12885-023-11545-3 (PMC10626706; doi:10.1186/s12885-023-11545-3)
Supplement: Supplementary file 1 — Supplementary Material 1 [file 12885_2023_11545_MOESM1_ESM.docx]

**Supplementary file**


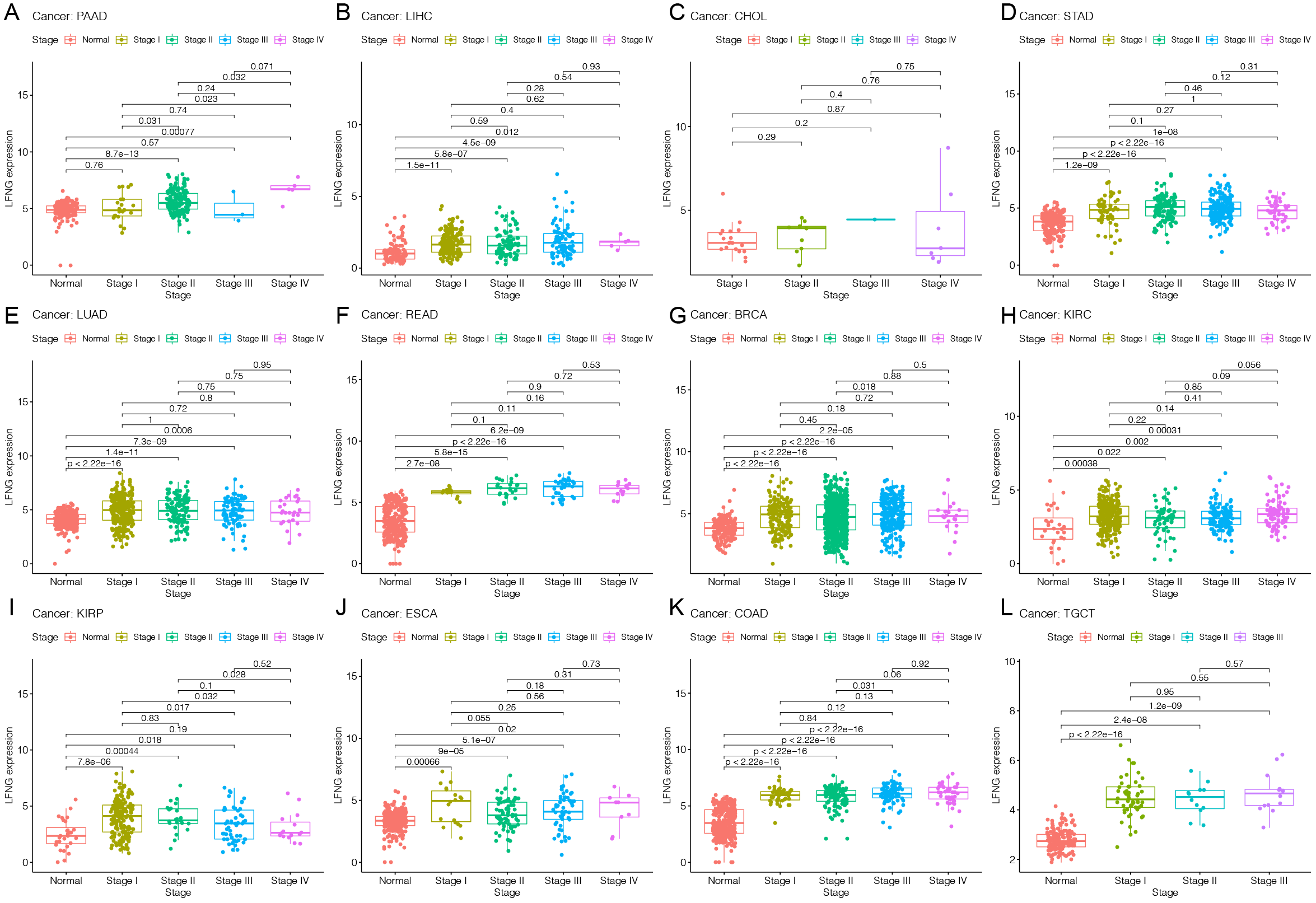


**Supplementary Fig. 1.** LFNG mRNA expression level in diverse tumor stages. A-L LFNG mRNA expression level in diverse stages of indicated tumor types from TCGA database. *p<0.05, **p<0.01, ***p<0.001, ****p<0.0001, ns: not significant.


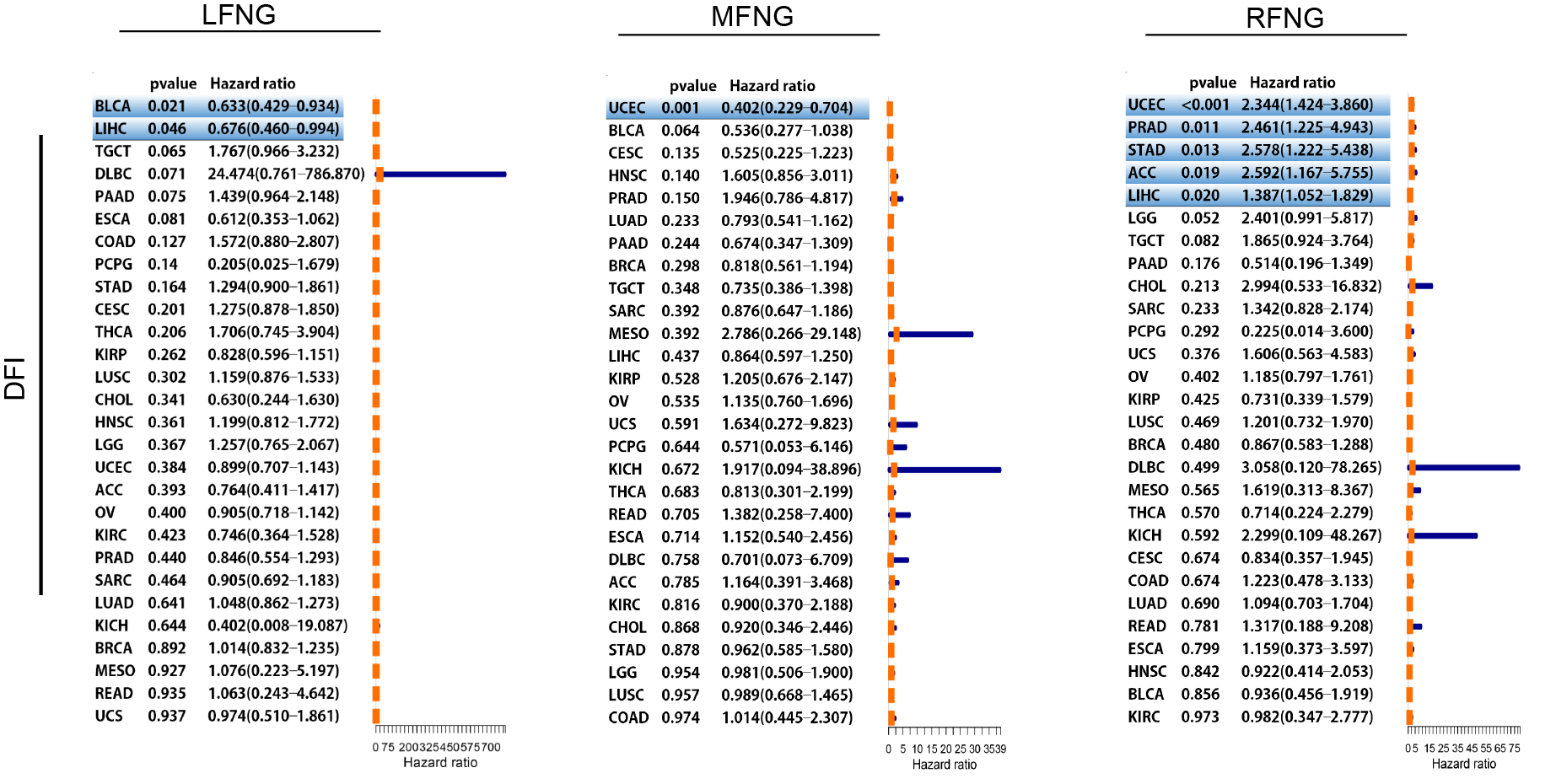


**Supplementary Fig. 2.** The Cox regression analysis of the relationship between LFNG, MFNG, and RFNG expression levels and disease-free interval (DFI) in different cancer types.

**
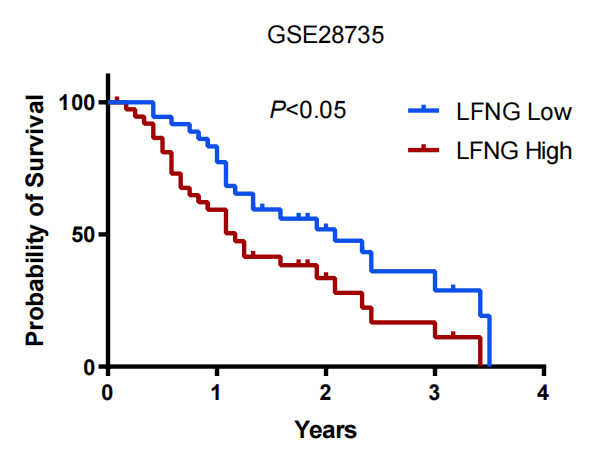
**

**Supplementary Fig. 3.** The verification of the prognostic discrimination of LFNG in pancreatic cancer by means of pancreatic cancer data set GSE28735 (Supplementary Table III).


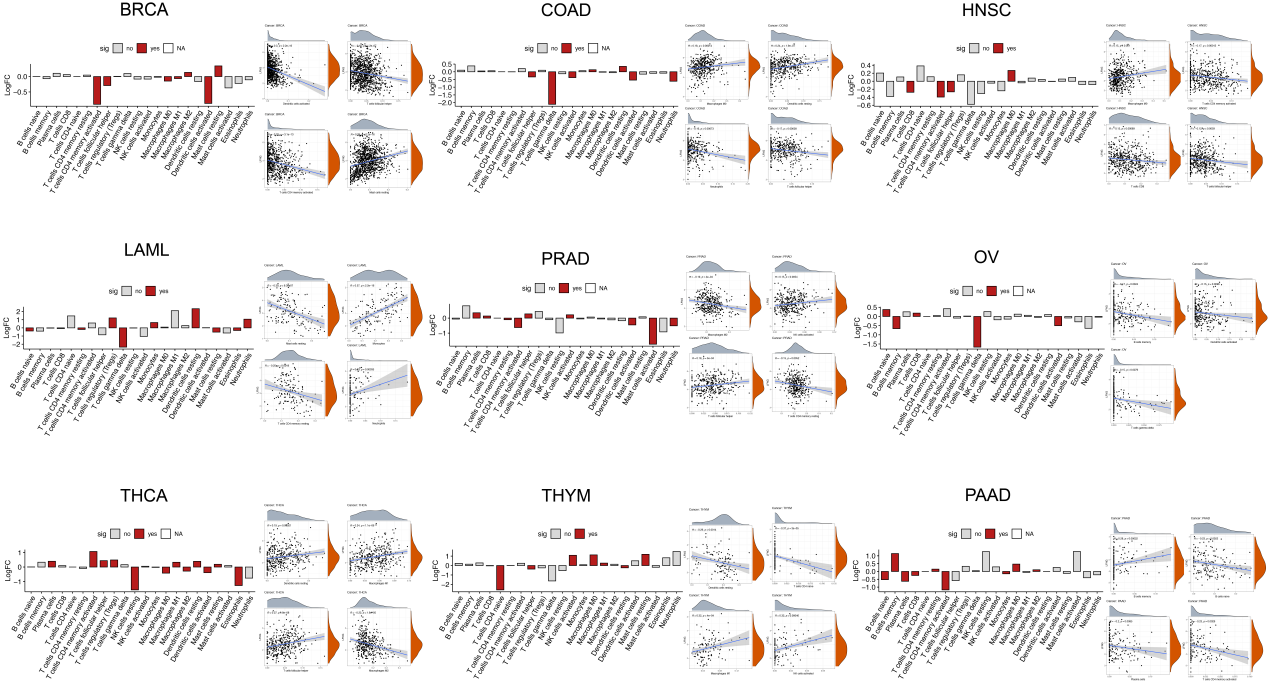


**Supplementary Fig. 4.** Association between LFNG expression level and infiltrated immune cells across tumors. *p<0.05, **p<0.01, ***p<0.001, ****p<0.0001, ns: not significant.


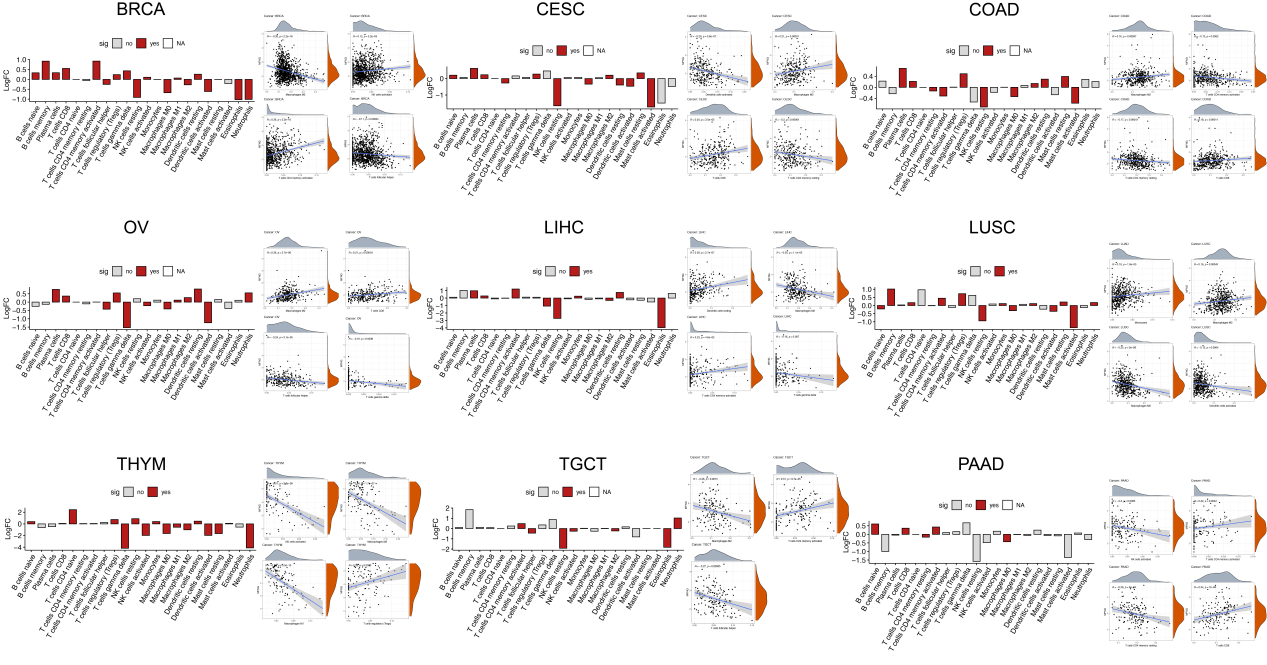


**Supplementary Fig. 5.** Association between MFNG expression level and infiltrated immune cells across tumors. *p<0.05, **p<0.01, ***p<0.001, ****p<0.0001, ns: not significant.


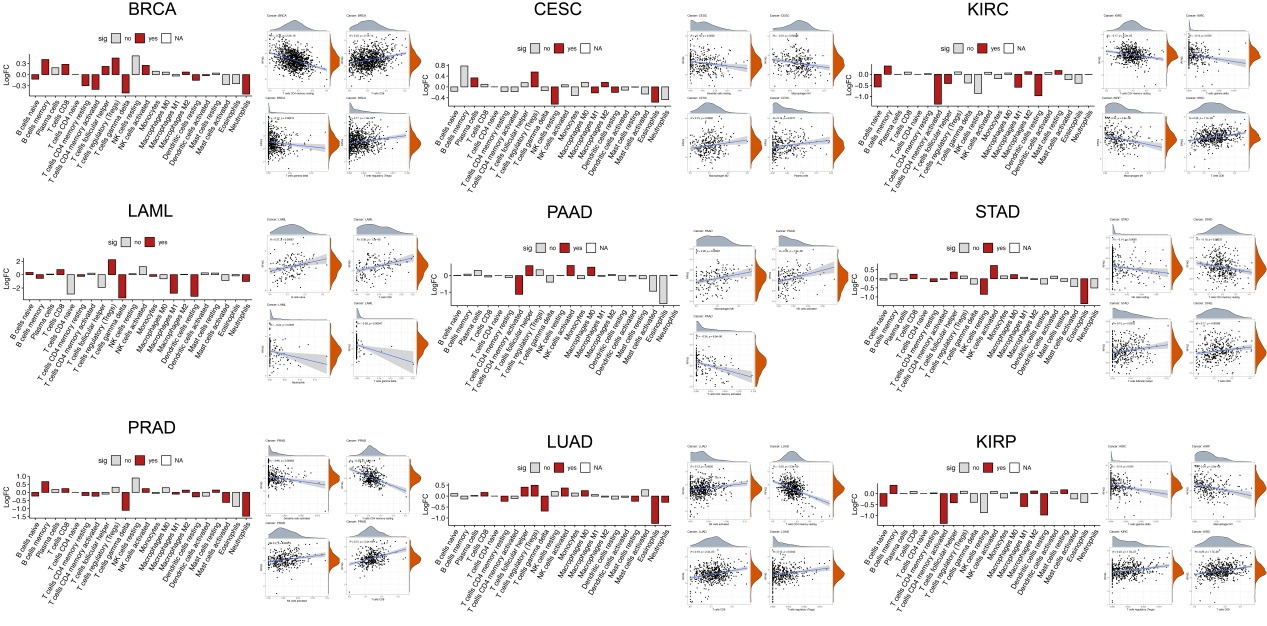


**Supplementary Fig. 6.** Association between RFNG expression level and infiltrated immune cells across tumors. *p<0.05, **p<0.01, ***p<0.001, ****p<0.0001, ns: not significant.


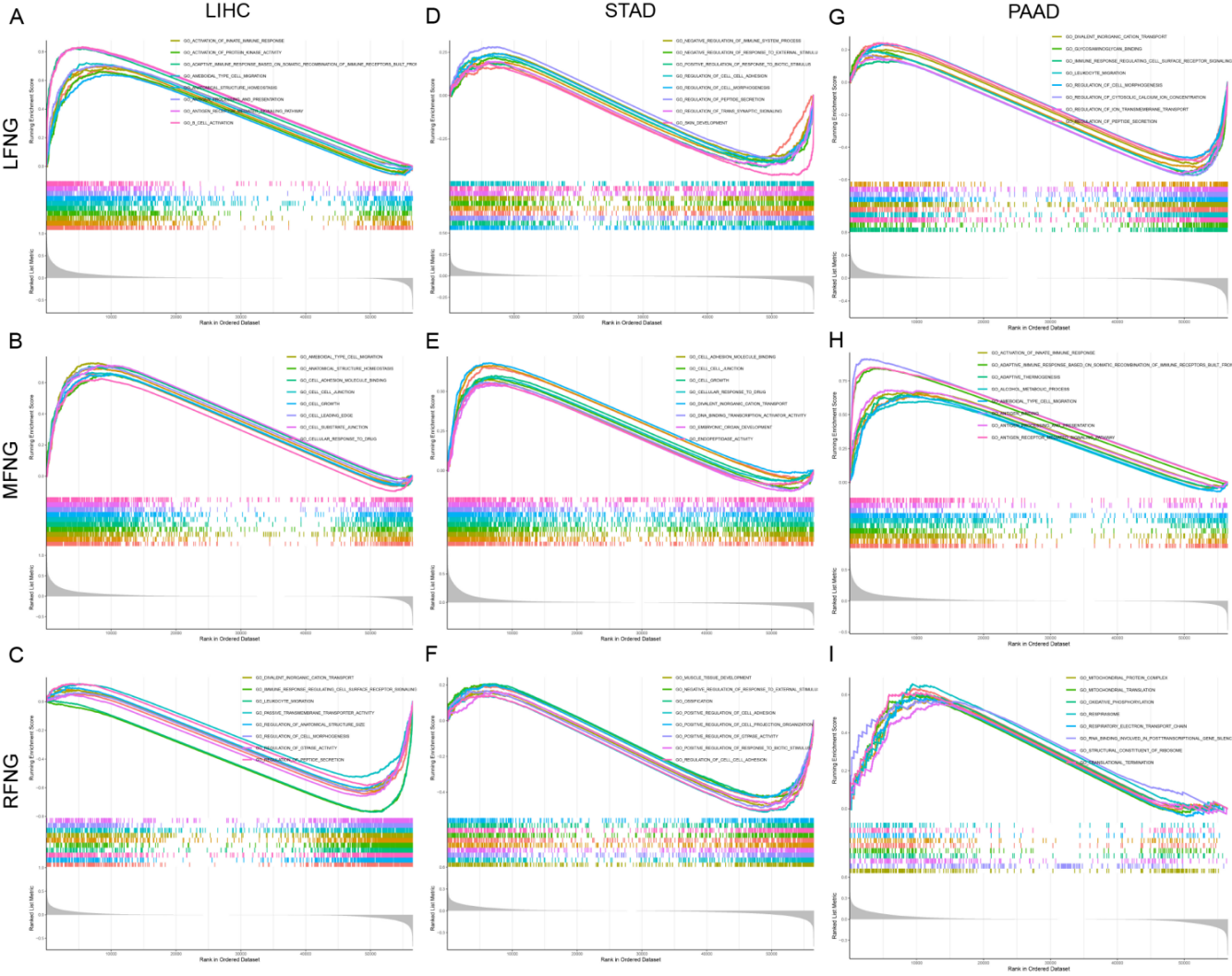


**Supplementary Fig. 7.** Correlation of LFNG, MFNG and RFNG expressions with GO terms by GSEA analysis. A-I. GSEA shows the top GO terms pathways correlated with LFNG, MFNG and RFNG expression in LIHC (A-C), STAD (D-F), PAAD (G-I), respectively.


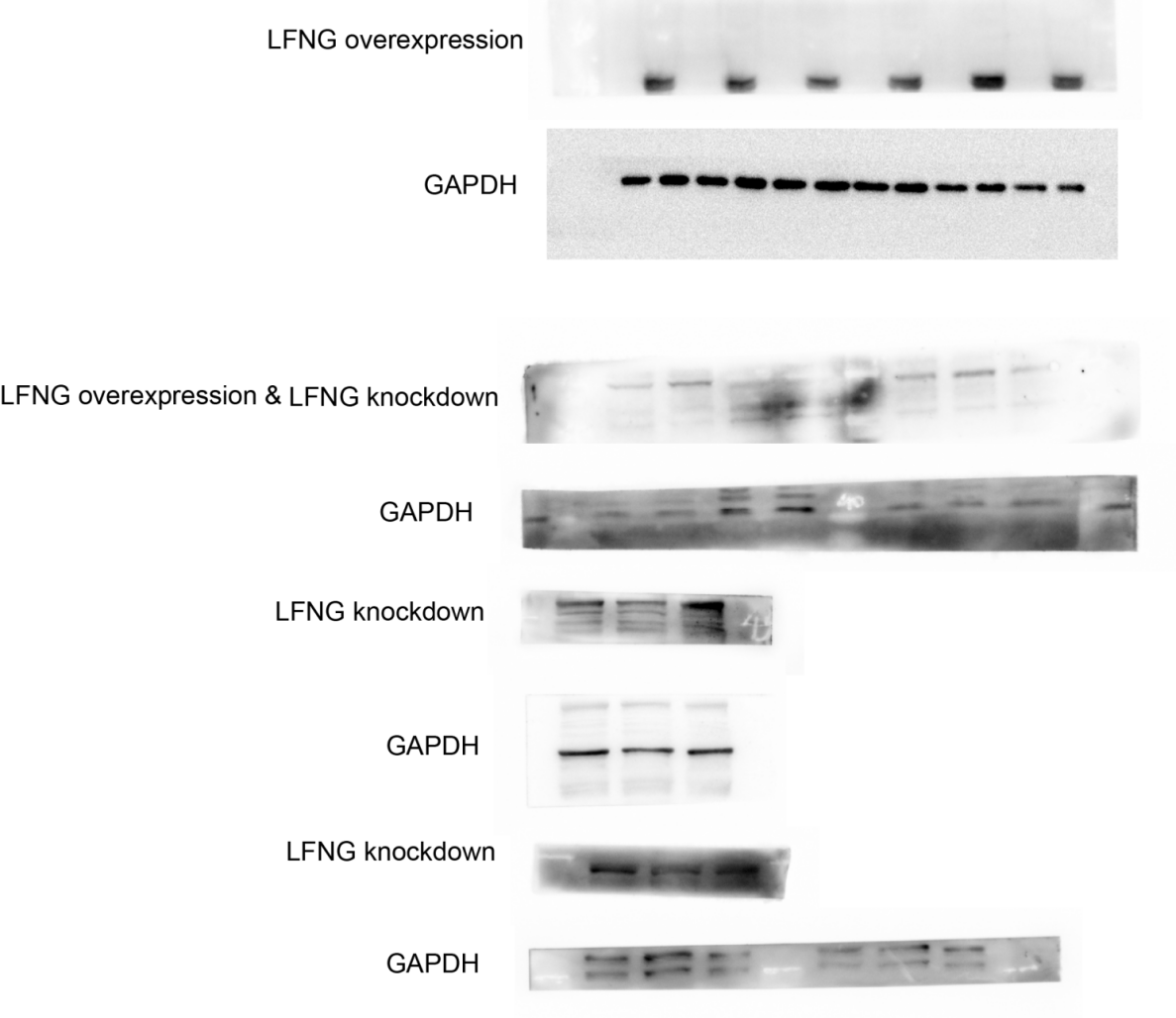


**Supplementary Fig. 8.** The original western blot image included the repeat results of LFNG overexpression and knockdown, respectively.

|  | cancer_type | LFNG | MFNG | RFNG |
| --- | --- | --- | --- | --- |
| 1 | ACC | 2.319 | 2.014 | 4.546 |
| 2 | BLCA | 3.65 | 2.046 | 5.214 |
| 3 | BRCA | 4.82 | 2.742 | 4.799 |
| 4 | CESC | 3.511 | 2.2705 | 4.7505 |
| 5 | CHOL | 3.1315 | 3.643 | 5.2145 |
| 6 | COAD | 6.0355 | 2.461 | 4.568 |
| 7 | DLBC | 2.878 | 5.887 | 4.135 |
| 8 | ESCA | 4.114 | 2.293 | 4.9695 |
| 9 | GBM | 5.967 | 4.073 | 5.166 |
| 10 | HNSC | 4.134 | 2.4805 | 4.486 |
| 11 | KICH | 1.2455 | 1.9395 | 3.71 |
| 12 | KIRC | 3.207 | 4.036 | 4.755 |
| 13 | KIRP | 3.704 | 2.727 | 5.47 |
| 14 | LAML | 3.754 | 5.985 | 4.584 |
| 15 | LGG | 5.784 | 3.45 | 5.139 |
| 16 | LIHC | 1.651 | 2.157 | 5.369 |
| 17 | LUAD | 4.929 | 3.37 | 4.929 |
| 18 | LUSC | 3.5325 | 2.636 | 4.7555 |
| 19 | MESO | 2.979 | 3.141 | 5.359 |
| 20 | OV | 2.888 | 2.043 | 5.404 |
| 21 | PAAD | 5.446 | 3.605 | 4.845 |
| 22 | PCPG | 2.8095 | 2.4065 | 5.0535 |
| 23 | PRAD | 5.383 | 2.121 | 5.3495 |
| 24 | READ | 6.068 | 2.354 | 4.496 |
| 25 | SARC | 3.352 | 3.265 | 5.2805 |
| 26 | SKCM | 2.511 | 2.452 | 5.202 |
| 27 | STAD | 4.9155 | 2.824 | 4.8685 |
| 28 | TGCT | 4.597 | 2.934 | 4.9325 |
| 29 | THCA | 2.596 | 2.677 | 4.964 |
| 30 | THYM | 3.129 | 4.831 | 5.265 |
| 31 | UCEC | 3.5 | 2.362 | 5.168 |
| 32 | UCS | 4.017 | 2.091 | 5.553 |
| 33 | UVM | 1.646 | 1.151 | 4.972 |

**Supplementary Table I :The expression median data of LFNG, MFNG and RFNG.**

| **Supplementary Table II : The PrognoScan data sets of LFNG, MFNG and RFNG for prognostic capabilities verification.** | | | | | | | | | | | | | | | | |
| --- | --- | --- | --- | --- | --- | --- | --- | --- | --- | --- | --- | --- | --- | --- | --- | --- |
|  | **DATASET** | **CANCER TYPE** | **SUBTYPE** | **ENDPOINT** | **COHORT** | **CONTRIBUTOR** | **ARRAY TYPE** | **PROBE ID** | **N** | **CUTPOINT** | **MINIMUM P-VALUE** | **CORRECTED P-VALUE** | **ln(HRhigh / HRlow)** | **COX P-VALUE** | **ln(HR)** | **HR [95% CIlow - CIupp]** |
| **LFNG** | **[GSE12417-GPL96](http://www.ncbi.nlm.nih.gov/geo/query/acc.cgi?acc=GSE12417)** | **Blood cancer** | **AML** | **Overall Survival** | **AMLCG (1999-2003)** | **Metzeler** | **HG-U133A** | **[215270_at](http://dna00.bio.kyutech.ac.jp/PrognoScan-cgi/PrognoScan.cgi?MODE=SHOW_GRAPH&TEST_NUM=13&DATA_POSTPROCESSING=None&TITLE=Prognostic+value of LFNG mRNA expression in Blood cancer&PROBE_ID=2014644)** | **163** | **0.47** | **0.002063** | **0.046459** | **-0.61** | **0.086037** | **-0.97** | **0.38 [0.12 - 1.15]** |
|  | **[GSE12417-GPL570](http://www.ncbi.nlm.nih.gov/geo/query/acc.cgi?acc=GSE12417)** | **Blood cancer** | **AML** | **Overall Survival** | **AMLCG (2004)** | **Metzeler** | **HG-U133_Plus_2** | **[215270_at](http://dna00.bio.kyutech.ac.jp/PrognoScan-cgi/PrognoScan.cgi?TITLE=Prognostic+value of LFNG mRNA expression in Blood cancer&DATA_POSTPROCESSING=None&TEST_NUM=12&PROBE_ID=4024565&MODE=SHOW_GRAPH)** | **79** | **0.57** | **0.004159** | **0.081813** | **-0.89** | **0.008491** | **-2.11** | **0.12 [0.03 - 0.58]** |
|  | **[E-TABM-346](ftp://ftp.ebi.ac.uk/pub/databases/microarray/data/experiment/TABM/E-TABM-346)** | **Blood cancer** | **DLBCL** | **Event Free Survival** | **GELA (1998-2000)** | **Jais** | **HG-U133A** | **[215270_at](http://dna00.bio.kyutech.ac.jp/PrognoScan-cgi/PrognoScan.cgi?TITLE=Prognostic+value of LFNG mRNA expression in Blood cancer&DATA_POSTPROCESSING=None&TEST_NUM=9&PROBE_ID=2014644&MODE=SHOW_GRAPH)** | **53** | **0.6** | **0.004027** | **0.079754** | **0.96** | **0.046762** | **0.65** | **1.91 [1.01 - 3.62]** |
|  | **[GSE2658](http://www.ncbi.nlm.nih.gov/geo/query/acc.cgi?acc=GSE2658)** | **Blood cancer** | **Multiple myeloma** | **Disease Specific Survival** | **Arkansas** | **Zhan** | **HG-U133_Plus_2** | **[228762_at](http://dna00.bio.kyutech.ac.jp/PrognoScan-cgi/PrognoScan.cgi?TITLE=Prognostic+value of LFNG mRNA expression in Blood cancer&DATA_POSTPROCESSING=None&TEST_NUM=56&PROBE_ID=4038017&MODE=SHOW_GRAPH)** | **559** | **0.36** | **0.003319** | **0.068334** | **-0.58** | **0.004637** | **-0.49** | **0.61 [0.43 - 0.86]** |
|  | **[GSE4271-GPL97](http://www.ncbi.nlm.nih.gov/geo/query/acc.cgi?acc=GSE4271)** | **Brain cancer** | **Astrocytoma** | **Overall Survival** | **MDA** | **Phillips** | **HG-U133B** | **[228762_at](http://dna00.bio.kyutech.ac.jp/PrognoScan-cgi/PrognoScan.cgi?TITLE=Prognostic+value of LFNG mRNA expression in Brain cancer&DATA_POSTPROCESSING=None&TEST_NUM=22&PROBE_ID=3006453&MODE=SHOW_GRAPH)** | **77** | **0.9** | **0.000001** | **0.000051** | **1.89** | **0.625861** | **0.06** | **1.06 [0.84 - 1.33]** |
|  | **[GSE7696](http://www.ncbi.nlm.nih.gov/geo/query/acc.cgi?acc=GSE7696)** | **Brain cancer** | **Glioblastoma** | **Overall Survival** | **Lausanne** | **Murat** | **HG-U133_Plus_2** | **[228762_at](http://dna00.bio.kyutech.ac.jp/PrognoScan-cgi/PrognoScan.cgi?PROBE_ID=4038017&DATA_POSTPROCESSING=None&TEST_NUM=36&TITLE=Prognostic+value of LFNG mRNA expression in Brain cancer&MODE=SHOW_GRAPH)** | **70** | **0.34** | **0.001489** | **0.035512** | **0.94** | **0.024087** | **0.48** | **1.61 [1.06 - 2.44]** |
|  | **[GSE4412-GPL96](http://www.ncbi.nlm.nih.gov/geo/query/acc.cgi?acc=GSE4412)** | **Brain cancer** | **Glioma** | **Overall Survival** | **UCLA (1996-2003)** | **Freije** | **HG-U133A** | **[215270_at](http://dna00.bio.kyutech.ac.jp/PrognoScan-cgi/PrognoScan.cgi?PROBE_ID=2014644&DATA_POSTPROCESSING=None&TEST_NUM=73&TITLE=Prognostic+value of LFNG mRNA expression in Brain cancer&MODE=SHOW_GRAPH)** | **74** | **0.47** | **0.017946** | **0.248095** | **-0.68** | **0.037463** | **-0.38** | **0.68 [0.48 - 0.98]** |
|  | **[GSE12276](http://www.ncbi.nlm.nih.gov/geo/query/acc.cgi?acc=GSE12276)** | **Breast cancer** |  | **Relapse Free Survival** | **EMC** | **Bos** | **HG-U133_Plus_2** | **[228762_at](http://dna00.bio.kyutech.ac.jp/PrognoScan-cgi/PrognoScan.cgi?PROBE_ID=4038017&DATA_POSTPROCESSING=None&TEST_NUM=89&TITLE=Prognostic+value of LFNG mRNA expression in Breast cancer&MODE=SHOW_GRAPH)** | **204** | **0.43** | **0.004172** | **0.082027** | **-0.4** | **0.034922** | **-0.14** | **0.87 [0.77 - 0.99]** |
|  | **[GSE9893](http://www.ncbi.nlm.nih.gov/geo/query/acc.cgi?acc=GSE9893)** | **Breast cancer** |  | **Overall Survival** | **Montpellier, Bordeaux, Turin (1989-2001)** | **Chanrion** | **MLRG Human 21K V12.0** | **[14336](http://dna00.bio.kyutech.ac.jp/PrognoScan-cgi/PrognoScan.cgi?MODE=SHOW_GRAPH&PROBE_ID=14014336&DATA_POSTPROCESSING=None&TITLE=Prognostic+value of LFNG mRNA expression in Breast cancer&TEST_NUM=55)** | **155** | **0.19** | **0.002722** | **0.058234** | **-0.85** | **0.036241** | **-0.18** | **0.83 [0.70 - 0.99]** |
|  | **[GSE2034](http://www.ncbi.nlm.nih.gov/geo/query/acc.cgi?acc=GSE2034)** | **Breast cancer** |  | **Distant Metastasis Free Survival** | **Rotterdam (1980-1995)** | **Wang** | **HG-U133A** | **[215270_at](http://dna00.bio.kyutech.ac.jp/PrognoScan-cgi/PrognoScan.cgi?TITLE=Prognostic+value of LFNG mRNA expression in Breast cancer&DATA_POSTPROCESSING=None&TEST_NUM=35&PROBE_ID=2014644&MODE=SHOW_GRAPH)** | **286** | **0.35** | **0.004718** | **0.09041** | **-0.55** | **0.026285** | **-0.31** | **0.74 [0.56 - 0.96]** |
|  | **[GSE17536](http://www.ncbi.nlm.nih.gov/geo/query/acc.cgi?acc=GSE17536)** | **Colorectal cancer** |  | **Disease Free Survival** | **MCC** | **Smith** | **HG-U133_Plus_2** | **[215270_at](http://dna00.bio.kyutech.ac.jp/PrognoScan-cgi/PrognoScan.cgi?TITLE=Prognostic+value of LFNG mRNA expression in Colorectal cancer&DATA_POSTPROCESSING=None&TEST_NUM=81&PROBE_ID=4024565&MODE=SHOW_GRAPH)** | **145** | **0.3** | **0.00542** | **0.10083** | **-0.91** | **0.013765** | **-1.73** | **0.18 [0.05 - 0.70]** |
|  | **[GSE17536](http://www.ncbi.nlm.nih.gov/geo/query/acc.cgi?acc=GSE17536)** | **Colorectal cancer** |  | **Overall Survival** | **MCC** | **Smith** | **HG-U133_Plus_2** | **[215270_at](http://dna00.bio.kyutech.ac.jp/PrognoScan-cgi/PrognoScan.cgi?TITLE=Prognostic+value of LFNG mRNA expression in Colorectal cancer&DATA_POSTPROCESSING=None&TEST_NUM=80&PROBE_ID=4024565&MODE=SHOW_GRAPH)** | **177** | **0.33** | **0.001957** | **0.044491** | **-0.72** | **0.010895** | **-1.19** | **0.30 [0.12 - 0.76]** |
|  | **[GSE17536](http://www.ncbi.nlm.nih.gov/geo/query/acc.cgi?acc=GSE17536)** | **Colorectal cancer** |  | **Disease Specific Survival** | **MCC** | **Smith** | **HG-U133_Plus_2** | **[215270_at](http://dna00.bio.kyutech.ac.jp/PrognoScan-cgi/PrognoScan.cgi?PROBE_ID=4024565&DATA_POSTPROCESSING=None&TEST_NUM=82&TITLE=Prognostic+value of LFNG mRNA expression in Colorectal cancer&MODE=SHOW_GRAPH)** | **177** | **0.34** | **0.003857** | **0.077062** | **-0.77** | **0.013941** | **-1.33** | **0.26 [0.09 - 0.76]** |
|  | **[GSE17537](http://www.ncbi.nlm.nih.gov/geo/query/acc.cgi?acc=GSE17537)** | **Colorectal cancer** |  | **Overall Survival** | **VMC** | **Smith** | **HG-U133_Plus_2** | **[228762_at](http://dna00.bio.kyutech.ac.jp/PrognoScan-cgi/PrognoScan.cgi?PROBE_ID=4038017&DATA_POSTPROCESSING=None&TEST_NUM=83&TITLE=Prognostic+value of LFNG mRNA expression in Colorectal cancer&MODE=SHOW_GRAPH)** | **55** | **0.18** | **0.012129** | **0.186502** | **-1.12** | **0.022524** | **-0.92** | **0.40 [0.18 - 0.88]** |
|  | **[GSE11595](http://www.ncbi.nlm.nih.gov/geo/query/acc.cgi?acc=GSE11595)** | **Esophagus cancer** | **Adenocarcinoma** | **Overall Survival** | **Sutton** | **Giddings** | **CRUKDMF_22K_v1.0.0** | **[40887](http://dna00.bio.kyutech.ac.jp/PrognoScan-cgi/PrognoScan.cgi?MODE=SHOW_GRAPH&PROBE_ID=8004624&DATA_POSTPROCESSING=None&TITLE=Prognostic+value of LFNG mRNA expression in Esophagus cancer&TEST_NUM=45)** | **34** | **0.12** | **0.000027** | **0.001082** | **-2.33** | **0.082038** | **-1.27** | **0.28 [0.07 - 1.18]** |
|  | **[GSE13213](http://www.ncbi.nlm.nih.gov/geo/query/acc.cgi?acc=GSE13213)** | **Lung cancer** | **Adenocarcinoma** | **Overall Survival** | **Nagoya (1995-1999, 2002-2004)** | **Tomida** | **G4112F** | **[A_23_P434518](http://dna00.bio.kyutech.ac.jp/PrognoScan-cgi/PrognoScan.cgi?MODE=SHOW_GRAPH&TEST_NUM=62&DATA_POSTPROCESSING=None&TITLE=Prognostic+value of LFNG mRNA expression in Lung cancer&PROBE_ID=17014070)** | **117** | **0.27** | **0.000008** | **0.000348** | **-1.22** | **0.000192** | **-0.93** | **0.39 [0.24 - 0.64]** |
|  | **[jacob-00182-UM](https://array.nci.nih.gov/caarray/project/jacob-00182)** | **Lung cancer** | **Adenocarcinoma** | **Overall Survival** | **UM** | **Shedden** | **HG-U133A** | **[215270_at](http://dna00.bio.kyutech.ac.jp/PrognoScan-cgi/PrognoScan.cgi?TITLE=Prognostic+value of LFNG mRNA expression in Lung cancer&DATA_POSTPROCESSING=None&TEST_NUM=3&PROBE_ID=2014644&MODE=SHOW_GRAPH)** | **178** | **0.84** | **0.001142** | **0.02848** | **0.77** | **0.227146** | **0.22** | **1.25 [0.87 - 1.78]** |
|  | **[DUKE-OC](http://data.cgt.duke.edu/oncogene.php)** | **Ovarian cancer** |  | **Overall Survival** | **Duke** | **Bild** | **HG-U133A** | **[215270_at](http://dna00.bio.kyutech.ac.jp/PrognoScan-cgi/PrognoScan.cgi?MODE=SHOW_GRAPH&TEST_NUM=53&DATA_POSTPROCESSING=None&TITLE=Prognostic+value of LFNG mRNA expression in Ovarian cancer&PROBE_ID=2014644)** | **133** | **0.8** | **0.002219** | **0.049308** | **0.81** | **0.007961** | **3.71** | **40.71 [2.64 - 628.90]** |
| **RFNG** | **[GSE12276](http://www.ncbi.nlm.nih.gov/geo/query/acc.cgi?acc=GSE12276)** | **Breast cancer** |  | **Relapse Free Survival** | **EMC** | **Bos** | **HG-U133_Plus_2** | **[212968_at](http://dna00.bio.kyutech.ac.jp/PrognoScan-cgi/PrognoScan.cgi?DATA_POSTPROCESSING=None&MODE=SHOW_GRAPH&TEST_NUM=89&PROBE_ID=4022272&TITLE=Prognostic+value of RFNG mRNA expression in Breast cancer)** | **204** | **0.19** | **0.000024** | **0.000993** | **-0.76** | **0.020115** | **-0.48** | **0.62 [0.41 - 0.93]** |
|  | **[GSE9893](http://www.ncbi.nlm.nih.gov/geo/query/acc.cgi?acc=GSE9893)** | **Breast cancer** |  | **Overall Survival** | **Montpellier, Bordeaux, Turin (1989-2001)** | **Chanrion** | **MLRG Human 21K V12.0** | **[2101](http://dna00.bio.kyutech.ac.jp/PrognoScan-cgi/PrognoScan.cgi?TEST_NUM=55&PROBE_ID=14002101&TITLE=Prognostic+value of RFNG mRNA expression in Breast cancer&DATA_POSTPROCESSING=None&MODE=SHOW_GRAPH)** | **155** | **0.33** | **0.000113** | **0.003892** | **-1.03** | **0.003798** | **-0.69** | **0.50 [0.31 - 0.80]** |
|  | **[GSE14333](http://www.ncbi.nlm.nih.gov/geo/query/acc.cgi?acc=GSE14333)** | **Colorectal cancer** |  | **Disease Free Survival** | **Melbourne** | **Jorissen** | **HG-U133_Plus_2** | **[212968_at](http://dna00.bio.kyutech.ac.jp/PrognoScan-cgi/PrognoScan.cgi?TITLE=Prognostic+value of RFNG mRNA expression in Colorectal cancer&PROBE_ID=4022272&TEST_NUM=68&MODE=SHOW_GRAPH&DATA_POSTPROCESSING=None)** | **226** | **0.9** | **0.004729** | **0.090582** | **0.96** | **0.045126** | **0.69** | **2.00 [1.02 - 3.94]** |
|  | **[GSE13213](http://www.ncbi.nlm.nih.gov/geo/query/acc.cgi?acc=GSE13213)** | **Lung cancer** | **Adenocarcinoma** | **Overall Survival** | **Nagoya (1995-1999, 2002-2004)** | **Tomida** | **G4112F** | **[A_24_P138912](http://dna00.bio.kyutech.ac.jp/PrognoScan-cgi/PrognoScan.cgi?DATA_POSTPROCESSING=None&MODE=SHOW_GRAPH&TEST_NUM=62&PROBE_ID=17019740&TITLE=Prognostic+value of RFNG mRNA expression in Lung cancer)** | **117** | **0.45** | **0.001549** | **0.036697** | **-0.9** | **0.008264** | **-0.8** | **0.45 [0.25 - 0.81]** |
|  | **[GSE31210](http://www.ncbi.nlm.nih.gov/geo/query/acc.cgi?acc=GSE31210)** | **Lung cancer** | **Adenocarcinoma** | **Relapse Free Survival** | **NCCRI** | **Okayama** | **HG-U133_Plus_2** | **[212968_at](http://dna00.bio.kyutech.ac.jp/PrognoScan-cgi/PrognoScan.cgi?DATA_POSTPROCESSING=None&MODE=SHOW_GRAPH&PROBE_ID=4022272&TEST_NUM=108&TITLE=Prognostic+value of RFNG mRNA expression in Lung cancer)** | **204** | **0.71** | **0.008015** | **0.136509** | **0.71** | **0.030938** | **1.15** | **3.16 [1.11 - 8.97]** |
|  | **[DUKE-OC](http://data.cgt.duke.edu/oncogene.php)** | **Ovarian cancer** |  | **Overall Survival** | **Duke** | **Bild** | **HG-U133A** | **[212968_at](http://dna00.bio.kyutech.ac.jp/PrognoScan-cgi/PrognoScan.cgi?TITLE=Prognostic+value of RFNG mRNA expression in Ovarian cancer&TEST_NUM=53&PROBE_ID=2012351&MODE=SHOW_GRAPH&DATA_POSTPROCESSING=None)** | **133** | **0.88** | **0.000288** | **0.008827** | **1.09** | **0.100062** | **0.28** | **1.33 [0.95 - 1.87]** |
|  | **[GSE19234](http://www.ncbi.nlm.nih.gov/projects/geo/query/acc.cgi?acc=GSE19234)** | **Skin cancer** | **Melanoma** | **Overall Survival** | **NYU** | **Bogunovic** | **HG-U133_Plus_2** | **[212968_at](http://dna00.bio.kyutech.ac.jp/PrognoScan-cgi/PrognoScan.cgi?DATA_POSTPROCESSING=None&MODE=SHOW_GRAPH&TEST_NUM=67&PROBE_ID=4022272&TITLE=Prognostic+value of RFNG mRNA expression in Skin cancer)** | **38** | **0.16** | **0.006126** | **0.110942** | **15.72** | **0.028633** | **1.25** | **3.50 [1.14 - 10.76]** |
| **MFNG** | **[GSE5122](http://www.ncbi.nlm.nih.gov/geo/query/acc.cgi?acc=GSE5122)** | **Blood cancer** | **AML** | **Overall Survival** | **San Diego** | **Raponi** | **HG-U133A** | **[204152_s_at](http://dna00.bio.kyutech.ac.jp/PrognoScan-cgi/PrognoScan.cgi?MODE=SHOW_GRAPH&DATA_POSTPROCESSING=None&TITLE=Prognostic+value of MFNG mRNA expression in Blood cancer&PROBE_ID=2003679&TEST_NUM=72)** | **58** | **0.1** | **0.008562** | **0.143557** | **1.29** | **0.04776** | **0.29** | **1.34 [1.00 - 1.79]** |
|  | **[GSE8970](http://www.ncbi.nlm.nih.gov/geo/query/acc.cgi?acc=GSE8970)** | **Blood cancer** | **AML** | **Overall Survival** | **San Diego** | **Raponi** | **HG-U133A** | **[213783_at](http://dna00.bio.kyutech.ac.jp/PrognoScan-cgi/PrognoScan.cgi?MODE=SHOW_GRAPH&TEST_NUM=37&PROBE_ID=2013163&TITLE=Prognostic+value of MFNG mRNA expression in Blood cancer&DATA_POSTPROCESSING=None)** | **34** | **0.47** | **0.002649** | **0.056969** | **-1.17** | **0.005344** | **-1.06** | **0.35 [0.17 - 0.73]** |
|  | **[GSE7696](http://www.ncbi.nlm.nih.gov/geo/query/acc.cgi?acc=GSE7696)** | **Brain cancer** | **Glioblastoma** | **Overall Survival** | **Lausanne** | **Murat** | **HG-U133_Plus_2** | **[213783_at](http://dna00.bio.kyutech.ac.jp/PrognoScan-cgi/PrognoScan.cgi?TITLE=Prognostic+value of MFNG mRNA expression in Brain cancer&PROBE_ID=4023084&DATA_POSTPROCESSING=None&TEST_NUM=36&MODE=SHOW_GRAPH)** | **70** | **0.11** | **0.000379** | **0.011183** | **-1.35** | **0.870865** | **0.12** | **1.13 [0.25 - 5.03]** |
|  | **[GSE3143](http://www.ncbi.nlm.nih.gov/geo/query/acc.cgi?acc=GSE3143)** | **Breast cancer** |  | **Overall Survival** | **Duke** | **Bild** | **HG-U95A** | **[41522_at](http://dna00.bio.kyutech.ac.jp/PrognoScan-cgi/PrognoScan.cgi?MODE=SHOW_GRAPH&TITLE=Prognostic+value of MFNG mRNA expression in Breast cancer&PROBE_ID=1011631&DATA_POSTPROCESSING=None&TEST_NUM=20)** | **158** | **0.9** | **0.000816** | **0.021488** | **1.18** | **0.345166** | **0.22** | **1.24 [0.79 - 1.96]** |
|  | **[GSE9893](http://www.ncbi.nlm.nih.gov/geo/query/acc.cgi?acc=GSE9893)** | **Breast cancer** |  | **Overall Survival** | **Montpellier, Bordeaux, Turin (1989-2001)** | **Chanrion** | **MLRG Human 21K V12.0** | **[15151](http://dna00.bio.kyutech.ac.jp/PrognoScan-cgi/PrognoScan.cgi?MODE=SHOW_GRAPH&PROBE_ID=14015151&TITLE=Prognostic+value of MFNG mRNA expression in Breast cancer&DATA_POSTPROCESSING=None&TEST_NUM=55)** | **155** | **0.18** | **0.000093** | **0.003277** | **-1.08** | **0.00174** | **-0.44** | **0.65 [0.49 - 0.85]** |
|  | **[GSE2034](http://www.ncbi.nlm.nih.gov/geo/query/acc.cgi?acc=GSE2034)** | **Breast cancer** |  | **Distant Metastasis Free Survival** | **Rotterdam (1980-1995)** | **Wang** | **HG-U133A** | **[204153_s_at](http://dna00.bio.kyutech.ac.jp/PrognoScan-cgi/PrognoScan.cgi?TITLE=Prognostic+value of MFNG mRNA expression in Breast cancer&PROBE_ID=2003680&DATA_POSTPROCESSING=None&TEST_NUM=35&MODE=SHOW_GRAPH)** | **286** | **0.28** | **0.000049** | **0.001878** | **-0.78** | **0.011029** | **-0.57** | **0.56 [0.36 - 0.88]** |
|  | **[GSE3494-GPL96](http://www.ncbi.nlm.nih.gov/geo/query/acc.cgi?acc=GSE3494)** | **Breast cancer** |  | **Disease Specific Survival** | **Uppsala (1987-1989)** | **Miller** | **HG-U133A** | **[204153_s_at](http://dna00.bio.kyutech.ac.jp/PrognoScan-cgi/PrognoScan.cgi?MODE=SHOW_GRAPH&PROBE_ID=2003680&TITLE=Prognostic+value of MFNG mRNA expression in Breast cancer&DATA_POSTPROCESSING=None&TEST_NUM=38)** | **236** | **0.24** | **0.001094** | **0.027474** | **-0.88** | **0.088946** | **-0.7** | **0.50 [0.22 - 1.11]** |
|  | **[GSE2990](http://www.ncbi.nlm.nih.gov/geo/query/acc.cgi?acc=GSE2990)** | **Breast cancer** |  | **Distant Metastasis Free Survival** | **Uppsala, Oxford** | **Sotiriou** | **HG-U133A** | **[204153_s_at](http://dna00.bio.kyutech.ac.jp/PrognoScan-cgi/PrognoScan.cgi?TITLE=Prognostic+value of MFNG mRNA expression in Breast cancer&PROBE_ID=2003680&DATA_POSTPROCESSING=None&TEST_NUM=90&MODE=SHOW_GRAPH)** | **125** | **0.86** | **0.015006** | **0.218069** | **-15.32** | **0.03301** | **-1.42** | **0.24 [0.07 - 0.89]** |
|  | **[GSE2990](http://www.ncbi.nlm.nih.gov/geo/query/acc.cgi?acc=GSE2990)** | **Breast cancer** |  | **Relapse Free Survival** | **Uppsala, Oxford** | **Sotiriou** | **HG-U133A** | **[204153_s_at](http://dna00.bio.kyutech.ac.jp/PrognoScan-cgi/PrognoScan.cgi?TEST_NUM=91&TITLE=Prognostic+value of MFNG mRNA expression in Breast cancer&PROBE_ID=2003680&DATA_POSTPROCESSING=None&MODE=SHOW_GRAPH)** | **125** | **0.86** | **0.001431** | **0.03436** | **-15.33** | **0.022333** | **-1.16** | **0.31 [0.12 - 0.85]** |
|  | **[GSE17536](http://www.ncbi.nlm.nih.gov/geo/query/acc.cgi?acc=GSE17536)** | **Colorectal cancer** |  | **Overall Survival** | **MCC** | **Smith** | **HG-U133_Plus_2** | **[213783_at](http://dna00.bio.kyutech.ac.jp/PrognoScan-cgi/PrognoScan.cgi?MODE=SHOW_GRAPH&DATA_POSTPROCESSING=None&PROBE_ID=4023084&TITLE=Prognostic+value of MFNG mRNA expression in Colorectal cancer&TEST_NUM=80)** | **177** | **0.51** | **0.000394** | **0.01156** | **-0.86** | **0.00187** | **-1.85** | **0.16 [0.05 - 0.50]** |
|  | **[GSE17536](http://www.ncbi.nlm.nih.gov/geo/query/acc.cgi?acc=GSE17536)** | **Colorectal cancer** |  | **Disease Specific Survival** | **MCC** | **Smith** | **HG-U133_Plus_2** | **[213783_at](http://dna00.bio.kyutech.ac.jp/PrognoScan-cgi/PrognoScan.cgi?MODE=SHOW_GRAPH&TEST_NUM=82&TITLE=Prognostic+value of MFNG mRNA expression in Colorectal cancer&PROBE_ID=4023084&DATA_POSTPROCESSING=None)** | **177** | **0.51** | **0.001782** | **0.041194** | **-0.87** | **0.003553** | **-2.02** | **0.13 [0.03 - 0.52]** |
|  | **[GSE17537](http://www.ncbi.nlm.nih.gov/geo/query/acc.cgi?acc=GSE17537)** | **Colorectal cancer** |  | **Overall Survival** | **VMC** | **Smith** | **HG-U133_Plus_2** | **[204153_s_at](http://dna00.bio.kyutech.ac.jp/PrognoScan-cgi/PrognoScan.cgi?MODE=SHOW_GRAPH&PROBE_ID=4013601&TITLE=Prognostic+value of MFNG mRNA expression in Colorectal cancer&DATA_POSTPROCESSING=None&TEST_NUM=83)** | **55** | **0.55** | **0.004402** | **0.085587** | **1.3** | **0.038983** | **1.57** | **4.80 [1.08 - 21.27]** |
|  | **[GSE17537](http://www.ncbi.nlm.nih.gov/geo/query/acc.cgi?acc=GSE17537)** | **Colorectal cancer** |  | **Overall Survival** | **VMC** | **Smith** | **HG-U133_Plus_2** | **[213783_at](http://dna00.bio.kyutech.ac.jp/PrognoScan-cgi/PrognoScan.cgi?MODE=SHOW_GRAPH&TEST_NUM=83&TITLE=Prognostic+value of MFNG mRNA expression in Colorectal cancer&PROBE_ID=4023084&DATA_POSTPROCESSING=None)** | **55** | **0.24** | **0.00057** | **0.015846** | **-1.44** | **0.025979** | **-3.45** | **0.03 [0.00 - 0.66]** |
|  | **[GSE17537](http://www.ncbi.nlm.nih.gov/geo/query/acc.cgi?acc=GSE17537)** | **Colorectal cancer** |  | **Disease Free Survival** | **VMC** | **Smith** | **HG-U133_Plus_2** | **[213783_at](http://dna00.bio.kyutech.ac.jp/PrognoScan-cgi/PrognoScan.cgi?PROBE_ID=4023084&TITLE=Prognostic+value of MFNG mRNA expression in Colorectal cancer&DATA_POSTPROCESSING=None&TEST_NUM=84&MODE=SHOW_GRAPH)** | **55** | **0.36** | **0.012781** | **0.193876** | **-1.15** | **0.039751** | **-3.23** | **0.04 [0.00 - 0.86]** |
|  | **[GSE22138](http://www.ncbi.nlm.nih.gov/geo/query/acc.cgi?acc=GSE22138)** | **Eye cancer** | **Uveal melanoma** | **Distant Metastasis Free Survival** | **BRCIC** | **Laurent** | **HG-U133_Plus_2** | **[204152_s_at](http://dna00.bio.kyutech.ac.jp/PrognoScan-cgi/PrognoScan.cgi?TEST_NUM=107&PROBE_ID=4013600&TITLE=Prognostic+value of MFNG mRNA expression in Eye cancer&DATA_POSTPROCESSING=None&MODE=SHOW_GRAPH)** | **63** | **0.52** | **0.003278** | **0.067646** | **-1.03** | **0.006319** | **-40.4** | **0.00 [0.00 - 0.00]** |
|  | **[GSE2837](http://www.ncbi.nlm.nih.gov/geo/query/acc.cgi?acc=GSE2837)** | **Head and neck cancer** | **Squamous cell carcinoma** | **Relapse Free Survival** | **VUMC, VAMC, UTMDACC (1992-2005)** | **Chung** | **U133_X3P** | **[g4505158_3p_a_at](http://dna00.bio.kyutech.ac.jp/PrognoScan-cgi/PrognoScan.cgi?MODE=SHOW_GRAPH&PROBE_ID=12011944&TITLE=Prognostic+value of MFNG mRNA expression in Head and neck cancer&DATA_POSTPROCESSING=None&TEST_NUM=52)** | **28** | **0.14** | **0.001244** | **0.030585** | **-1.75** | **0.12288** | **-0.76** | **0.47 [0.18 - 1.23]** |
|  | **[jacob-00182-CANDF](https://array.nci.nih.gov/caarray/project/jacob-00182)** | **Lung cancer** | **Adenocarcinoma** | **Overall Survival** | **CAN/DF** | **Shedden** | **HG-U133A** | **[204153_s_at](http://dna00.bio.kyutech.ac.jp/PrognoScan-cgi/PrognoScan.cgi?MODE=SHOW_GRAPH&PROBE_ID=2003680&TITLE=Prognostic+value of MFNG mRNA expression in Lung cancer&DATA_POSTPROCESSING=None&TEST_NUM=5)** | **82** | **0.13** | **0.00049** | **0.013924** | **-1.33** | **0.99449** | **0** | **1.00 [0.27 - 3.63]** |
|  | **[GSE13213](http://www.ncbi.nlm.nih.gov/geo/query/acc.cgi?acc=GSE13213)** | **Lung cancer** | **Adenocarcinoma** | **Overall Survival** | **Nagoya (1995-1999, 2002-2004)** | **Tomida** | **G4112F** | **[A_23_P103104](http://dna00.bio.kyutech.ac.jp/PrognoScan-cgi/PrognoScan.cgi?TEST_NUM=62&TITLE=Prognostic+value of MFNG mRNA expression in Lung cancer&PROBE_ID=17000246&DATA_POSTPROCESSING=None&MODE=SHOW_GRAPH)** | **117** | **0.44** | **0.015678** | **0.225114** | **-0.69** | **0.037147** | **-0.37** | **0.69 [0.49 - 0.98]** |
|  | **[GSE13213](http://www.ncbi.nlm.nih.gov/geo/query/acc.cgi?acc=GSE13213)** | **Lung cancer** | **Adenocarcinoma** | **Overall Survival** | **Nagoya (1995-1999, 2002-2004)** | **Tomida** | **G4112F** | **[A_24_P224926](http://dna00.bio.kyutech.ac.jp/PrognoScan-cgi/PrognoScan.cgi?MODE=SHOW_GRAPH&DATA_POSTPROCESSING=None&PROBE_ID=17021905&TITLE=Prognostic+value of MFNG mRNA expression in Lung cancer&TEST_NUM=62)** | **117** | **0.1** | **0.006327** | **0.113765** | **-1.02** | **0.049752** | **-0.34** | **0.71 [0.50 - 1.00]** |
|  | **[GSE31210](http://www.ncbi.nlm.nih.gov/geo/query/acc.cgi?acc=GSE31210)** | **Lung cancer** | **Adenocarcinoma** | **Relapse Free Survival** | **NCCRI** | **Okayama** | **HG-U133_Plus_2** | **[213783_at](http://dna00.bio.kyutech.ac.jp/PrognoScan-cgi/PrognoScan.cgi?MODE=SHOW_GRAPH&TEST_NUM=108&PROBE_ID=4023084&TITLE=Prognostic+value of MFNG mRNA expression in Lung cancer&DATA_POSTPROCESSING=None)** | **204** | **0.46** | **0.001122** | **0.028073** | **0.97** | **0.108386** | **0.27** | **1.31 [0.94 - 1.84]** |
|  | **[GSE8894](http://www.ncbi.nlm.nih.gov/geo/query/acc.cgi?acc=GSE8894)** | **Lung cancer** | **NSCLC** | **Relapse Free Survival** | **Seoul (1995-2005)** | **Lee** | **HG-U133_Plus_2** | **[204153_s_at](http://dna00.bio.kyutech.ac.jp/PrognoScan-cgi/PrognoScan.cgi?MODE=SHOW_GRAPH&TITLE=Prognostic+value of MFNG mRNA expression in Lung cancer&PROBE_ID=4013601&DATA_POSTPROCESSING=None&TEST_NUM=32)** | **138** | **0.48** | **0.004899** | **0.093137** | **-0.69** | **0.023825** | **-0.26** | **0.77 [0.61 - 0.97]** |
|  | **[DUKE-OC](http://data.cgt.duke.edu/oncogene.php)** | **Ovarian cancer** |  | **Overall Survival** | **Duke** | **Bild** | **HG-U133A** | **[204152_s_at](http://dna00.bio.kyutech.ac.jp/PrognoScan-cgi/PrognoScan.cgi?MODE=SHOW_GRAPH&TEST_NUM=53&TITLE=Prognostic+value of MFNG mRNA expression in Ovarian cancer&PROBE_ID=2003679&DATA_POSTPROCESSING=None)** | **133** | **0.82** | **0.001086** | **0.0273** | **0.88** | **0.008215** | **0.51** | **1.67 [1.14 - 2.45]** |
|  | **[DUKE-OC](http://data.cgt.duke.edu/oncogene.php)** | **Ovarian cancer** |  | **Overall Survival** | **Duke** | **Bild** | **HG-U133A** | **[213783_at](http://dna00.bio.kyutech.ac.jp/PrognoScan-cgi/PrognoScan.cgi?TEST_NUM=53&DATA_POSTPROCESSING=None&TITLE=Prognostic+value of MFNG mRNA expression in Ovarian cancer&PROBE_ID=2013163&MODE=SHOW_GRAPH)** | **133** | **0.63** | **0.000393** | **0.01154** | **0.82** | **0.005626** | **3.13** | **22.99 [2.50 - 211.46]** |
|  | **[E-DKFZ-1](ftp://ftp.ebi.ac.uk/pub/databases/microarray/data/experiment/DKFZ/E-DKFZ-1)** | **Renal cell carcinoma** |  | **Overall Survival** | **RZPD** | **Sueltmann** | **A-RZPD-20** | **[rzpd.de:huber1:Reporter:IMAGE:132656](http://dna00.bio.kyutech.ac.jp/PrognoScan-cgi/PrognoScan.cgi?MODE=SHOW_GRAPH&TEST_NUM=47&TITLE=Prognostic+value of MFNG mRNA expression in Renal cell carcinoma&PROBE_ID=10000280&DATA_POSTPROCESSING=None)** | **59** | **0.61** | **0.008057** | **0.137056** | **1.26** | **0.012015** | **1.92** | **6.79 [1.52 - 30.29]** |
|  | **[GSE19234](http://www.ncbi.nlm.nih.gov/projects/geo/query/acc.cgi?acc=GSE19234)** | **Skin cancer** | **Melanoma** | **Overall Survival** | **NYU** | **Bogunovic** | **HG-U133_Plus_2** | **[204153_s_at](http://dna00.bio.kyutech.ac.jp/PrognoScan-cgi/PrognoScan.cgi?MODE=SHOW_GRAPH&TEST_NUM=67&TITLE=Prognostic+value of MFNG mRNA expression in Skin cancer&PROBE_ID=4013601&DATA_POSTPROCESSING=None)** | **38** | **0.68** | **0.003096** | **0.064604** | **-2.41** | **0.024501** | **-0.94** | **0.39 [0.17 - 0.89]** |
|  | **[GSE30929](http://www.ncbi.nlm.nih.gov/geo/query/acc.cgi?acc=GSE30929)** | **Soft tissue cancer** | **Liposarcoma** | **Distant Recurrence Free Survival** | **MSKCC (1993-2008)** | **Gobble** | **HG-U133A** | **[204152_s_at](http://dna00.bio.kyutech.ac.jp/PrognoScan-cgi/PrognoScan.cgi?PROBE_ID=2003679&TITLE=Prognostic+value of MFNG mRNA expression in Soft tissue cancer&DATA_POSTPROCESSING=None&TEST_NUM=110&MODE=SHOW_GRAPH)** | **140** | **0.17** | **0.003701** | **0.074555** | **-0.94** | **0.006049** | **-2.06** | **0.13 [0.03 - 0.56]** |
|  | **[GSE30929](http://www.ncbi.nlm.nih.gov/geo/query/acc.cgi?acc=GSE30929)** | **Soft tissue cancer** | **Liposarcoma** | **Distant Recurrence Free Survival** | **MSKCC (1993-2008)** | **Gobble** | **HG-U133A** | **[204153_s_at](http://dna00.bio.kyutech.ac.jp/PrognoScan-cgi/PrognoScan.cgi?MODE=SHOW_GRAPH&DATA_POSTPROCESSING=None&TITLE=Prognostic+value of MFNG mRNA expression in Soft tissue cancer&PROBE_ID=2003680&TEST_NUM=110)** | **140** | **0.22** | **0.001316** | **0.032053** | **-0.94** | **0.003383** | **-1.27** | **0.28 [0.12 - 0.66]** |

| **Supplementary Table III :The pancreatic cancer data set GSE28735 for prognostic discrimination of LFNG in pancreatic cancer.** | | | |
| --- | --- | --- | --- |
| futime | fustat | LFNG | group |
| **3.5** | **1** | **4.03145** | **Low** |
| **3.5** | **1** | **3.8873** | **Low** |
| **3.416666667** | **1** | **3.90636** | **Low** |
| **3.166666667** | **0** | **4.24324** | **Low** |
| **3** | **1** | **3.76822** | **Low** |
| **2.416666667** | **1** | **4.11271** | **Low** |
| **2.333333333** | **1** | **4.03334** | **Low** |
| **2.333333333** | **0** | **4.17408** | **Low** |
| **2.333333333** | **0** | **4.02216** | **Low** |
| **2.333333333** | **0** | **4.1881** | **Low** |
| **2.333333333** | **0** | **3.39222** | **Low** |
| **2.083333333** | **1** | **4.22687** | **Low** |
| **2** | **0** | **4.19151** | **Low** |
| **1.916666667** | **1** | **3.83863** | **Low** |
| **1.833333333** | **0** | **3.79319** | **Low** |
| **1.75** | **0** | **3.50127** | **Low** |
| **1.583333333** | **1** | **4.25143** | **Low** |
| **1.416666667** | **0** | **4.07675** | **Low** |
| **1.416666667** | **0** | **3.8799** | **Low** |
| **1.333333333** | **1** | **4.03929** | **Low** |
| **1.333333333** | **0** | **3.67154** | **Low** |
| **1.333333333** | **1** | **3.68312** | **Low** |
| **1.166666667** | **1** | **3.86187** | **Low** |
| **1.083333333** | **1** | **3.95887** | **Low** |
| **1.083333333** | **1** | **3.90012** | **Low** |
| **1.083333333** | **1** | **4.03262** | **Low** |
| **1** | **1** | **3.74302** | **Low** |
| **1** | **1** | **4.12681** | **Low** |
| **0.916666667** | **0** | **4.17759** | **Low** |
| **0.916666667** | **1** | **4.21759** | **Low** |
| **0.833333333** | **1** | **4.17408** | **Low** |
| **0.833333333** | **0** | **4.2281** | **Low** |
| **0.75** | **1** | **3.94662** | **Low** |
| **0.583333333** | **1** | **4.17701** | **Low** |
| **0.416666667** | **1** | **3.92519** | **Low** |
| **0.416666667** | **1** | **4.14382** | **Low** |
| **3.416666667** | **1** | **4.31277** | **High** |
| **3.166666667** | **0** | **4.35969** | **High** |
| **3** | **1** | **4.6478** | **High** |
| **2.416666667** | **1** | **4.47479** | **High** |
| **2.333333333** | **1** | **4.72785** | **High** |
| **2.083333333** | **1** | **4.4023** | **High** |
| **2** | **0** | **4.83504** | **High** |
| **1.916666667** | **1** | **4.25952** | **High** |
| **1.833333333** | **0** | **4.96358** | **High** |
| **1.75** | **0** | **4.66985** | **High** |
| **1.75** | **0** | **4.51054** | **High** |
| **1.75** | **0** | **4.58515** | **High** |
| **1.583333333** | **1** | **4.30206** | **High** |
| **1.333333333** | **0** | **4.37657** | **High** |
| **1.25** | **1** | **4.50592** | **High** |
| **1.25** | **1** | **4.2767** | **High** |
| **1.166666667** | **1** | **4.53189** | **High** |
| **1.083333333** | **1** | **4.42376** | **High** |
| **1.083333333** | **1** | **4.35479** | **High** |
| **1.083333333** | **1** | **4.52588** | **High** |
| **0.916666667** | **1** | **4.49988** | **High** |
| **0.916666667** | **0** | **4.51991** | **High** |
| **0.833333333** | **1** | **4.70281** | **High** |
| **0.833333333** | **0** | **5.05112** | **High** |
| **0.75** | **1** | **4.38586** | **High** |
| **0.666666667** | **1** | **4.39237** | **High** |
| **0.666666667** | **1** | **4.73905** | **High** |
| **0.583333333** | **1** | **4.26156** | **High** |
| **0.583333333** | **1** | **4.69497** | **High** |
| **0.583333333** | **1** | **4.54386** | **High** |
| **0.5** | **1** | **4.28372** | **High** |
| **0.5** | **1** | **4.26987** | **High** |
| **0.416666667** | **1** | **4.35372** | **High** |
| **0.416666667** | **1** | **4.64419** | **High** |
| **0.333333333** | **1** | **4.42152** | **High** |
| **0.25** | **1** | **4.63205** | **High** |
| **0.166666667** | **1** | **4.34469** | **High** |
| **0.083333333** | **0** | **4.38639** | **High** |
| **0.083333333** | **0** | **4.27082** | **High** |

|  | **UniProtKB ID** | **Genes** |
| --- | --- | --- |
| 1 | UniProtKB:P46531 | NOTCH1 |
| 2 | UniProtKB:Q7Z3S9 | NOTCH2NLA |
| 3 | UniProtKB:Q99466 | NOTCH4 |
| 4 | UniProtKB:P0DPK4 | NOTCH2NLC |
| 5 | UniProtKB:Q9UM47 | NOTCH3 |
| 6 | UniProtKB:A0A096LNW5 | NOTCH2NLR |
| 7 | UniProtKB:P0DPK3 | NOTCH2NLB |
| 8 | UniProtKB:Q04721 | NOTCH2 |
| 9 | UniProtKB:Q9H488 | POFUT1 |
| 10 | UniProtKB:Q96K30 | RITA1 |
| 11 | UniProtKB:Q15334 | LLGL1 |
| 12 | UniProtKB:O43623 | SNAI2 |
| 13 | UniProtKB:Q5VV63 | ATRNL1 |
| 14 | ComplexPortal:CPX-330 | ccnc-cdk3_human |
| 15 | UniProtKB:Q8WW43 | APH1B |
| 16 | UniProtKB:A6XGL0 | YJEFN3 |
| 17 | UniProtKB:Q5TAB7 | RIPPLY2 |
| 18 | UniProtKB:Q5TA89 | HES5 |
| 19 | UniProtKB:P41743 | PRKCI |
| 20 | UniProtKB:P48436 | SOX9 |
| 21 | UniProtKB:Q12860 | CNTN1 |
| 22 | UniProtKB:Q9NSY1 | BMP2K |
| 23 | UniProtKB:P57105 | SYNJ2BP |
| 24 | UniProtKB:P40189 | IL6ST |
| 25 | UniProtKB:Q7Z6K4 | NRARP |
| 26 | UniProtKB:P78504 | JAG1 |
| 27 | UniProtKB:Q13573 | SNW1 |
| 28 | UniProtKB:P41182 | BCL6 |
| 29 | UniProtKB:P55285 | CDH6 |
| 30 | UniProtKB:Q8N5I2 | ARRDC1 |
| 31 | UniProtKB:Q8N5F7 | NKAP |
| 32 | UniProtKB:P05067 | APP |
| 33 | UniProtKB:P23769 | GATA2 |
| 34 | UniProtKB:Q8N9I9 | DTX3 |
| 35 | UniProtKB:Q9BUL8 | PDCD10 |
| 36 | UniProtKB:Q5T655 | CFAP58 |
| 37 | UniProtKB:Q07687 | DLX2 |
| 38 | UniProtKB:Q86YT6 | MIB1 |
| 39 | UniProtKB:P48745 | CCN3 |
| 40 | UniProtKB:P21549 | AGXT |
| 41 | UniProtKB:P31749 | AKT1 |
| 42 | UniProtKB:O76050 | NEURL1 |
| 43 | UniProtKB:Q9Y6I3 | EPN1 |
| 44 | UniProtKB:Q15475 | SIX1 |
| 45 | UniProtKB:Q15466 | NR0B2 |
| 46 | UniProtKB:O00587 | MFNG |
| 47 | UniProtKB:Q6UY11 | DLK2 |
| 48 | UniProtKB:Q8TDB6 | DTX3L |
| 49 | UniProtKB:P80370 | DLK1 |
| 50 | UniProtKB:Q00534 | CDK6 |
| 51 | UniProtKB:Q00526 | CDK3 |
| 52 | UniProtKB:P18075 | BMP7 |
| 53 | UniProtKB:Q6UWT2 | ENHO |
| 54 | UniProtKB:Q9BZM3 | GSX2 |
| 55 | UniProtKB:Q13618 | CUL3 |
| 56 | UniProtKB:Q86U44 | METTL3 |
| 57 | UniProtKB:Q9BWX5 | GATA5 |
| 58 | UniProtKB:Q2M2I8 | AAK1 |
| 59 | UniProtKB:Q9NZ42 | PSENEN |
| 60 | UniProtKB:Q86YV5 | PRAG1 |
| 61 | UniProtKB:Q8TER0 | SNED1 |
| 62 | UniProtKB:Q9UQ52 | CNTN6 |
| 63 | UniProtKB:Q99500 | S1PR3 |
| 64 | UniProtKB:P31249 | HOXD3 |
| 65 | UniProtKB:Q15063 | POSTN |

|  | **UniProtKB ID** | **Genes** |
| --- | --- | --- |
| 66 | UniProtKB:Q6Q0C0 | TRAF7 |
| 67 | UniProtKB:P09525 | ANXA4 |
| 68 | UniProtKB:Q9NWT6 | HIF1AN |
| 69 | UniProtKB:Q96RI1 | NR1H4 |
| 70 | UniProtKB:Q9GZL7 | WDR12 |
| 71 | UniProtKB:P12931 | SRC |
| 72 | UniProtKB:Q96TA1 | NIBAN2 |
| 73 | UniProtKB:Q9NYJ7 | DLL3 |
| 74 | UniProtKB:Q2M1K9 | ZNF423 |
| 75 | UniProtKB:P46527 | CDKN1B |
| 76 | UniProtKB:Q9BYE0 | HES7 |
| 77 | UniProtKB:P56177 | DLX1 |
| 78 | UniProtKB:A3KN83 | SBNO1 |
| 79 | UniProtKB:Q9HC29 | NOD2 |
| 80 | UniProtKB:Q92542 | NCSTN |
| 81 | UniProtKB:Q5SZJ8 | BEND6 |
| 82 | UniProtKB:P04628 | WNT1 |
| 83 | UniProtKB:Q96T37 | RBM15 |
| 84 | UniProtKB:Q92858 | ATOH1 |
| 85 | UniProtKB:O00548 | DLL1 |
| 86 | UniProtKB:Q9UPX6 | MINAR1 |
| 87 | UniProtKB:O94875 | SORBS2 |
| 88 | UniProtKB:O00321 | ETV2 |
| 89 | UniProtKB:O00308 | WWP2 |
| 90 | UniProtKB:Q8WU66 | TSPEAR |
| 91 | UniProtKB:Q8IZL2 | MAML2 |
| 92 | UniProtKB:P26678 | PLN |
| 93 | UniProtKB:P24863 | CCNC |
| 94 | UniProtKB:Q99727 | TIMP4 |
| 95 | UniProtKB:Q99958 | FOXC2 |
| 96 | UniProtKB:Q9Y618 | NCOR2 |
| 97 | UniProtKB:P51608 | MECP2 |
| 98 | UniProtKB:Q86Y01 | DTX1 |
| 99 | UniProtKB:P46937 | YAP1 |
| 100 | UniProtKB:P61812 | TGFB2 |
| 101 | UniProtKB:Q9HD90 | NEUROD4 |
| 102 | UniProtKB:P01589 | IL2RA |
| 103 | UniProtKB:P43355 | MAGEA1 |
| 104 | UniProtKB:Q6NW34 | NEPRO |
| 105 | UniProtKB:Q86UW9 | DTX2 |
| 106 | UniProtKB:P78536 | ADAM17 |
| 107 | UniProtKB:Q12948 | FOXC1 |
| 108 | UniProtKB:P15259 | PGAM2 |
| 109 | UniProtKB:P62079 | TSPAN5 |
| 110 | UniProtKB:Q9C0E4 | GRIP2 |
| 111 | UniProtKB:Q6P1M3 | LLGL2 |
| 112 | UniProtKB:Q12772 | SREBF2 |
| 113 | UniProtKB:Q969H0 | FBXW7 |
| 114 | UniProtKB:Q9BRJ9 | MESP1 |
| 115 | UniProtKB:Q9NV29 | TMEM100 |
| 116 | UniProtKB:O14713 | ITGB1BP1 |
| 117 | UniProtKB:P35680 | HNF1B |
| 118 | UniProtKB:P50553 | ASCL1 |
| 119 | UniProtKB:Q92585 | MAML1 |
| 120 | UniProtKB:O14672 | ADAM10 |
| 121 | UniProtKB:O15520 | FGF10 |
| 122 | UniProtKB:Q9Y264 | ANGPT4 |
| 123 | UniProtKB:Q16552 | IL17A |
| 124 | UniProtKB:Q9Y2E6 | DTX4 |
| 125 | UniProtKB:O75365 | PTP4A3 |
| 126 | UniProtKB:P37173 | TGFBR2 |
| 127 | UniProtKB:Q9NQQ7 | SLC35C2 |
| 128 | UniProtKB:Q9NR00 | TCIM |
| 129 | UniProtKB:P49715 | CEBPA |
| 130 | UniProtKB:Q96JK9 | MAML3 |

|  | **UniProtKB ID** | **Genes** |
| --- | --- | --- |
| 131 | UniProtKB:Q9H3F6 | KCTD10 |
| 132 | UniProtKB:P22460 | KCNA5 |
| 133 | UniProtKB:Q9BRN9 | TM2D3 |
| 134 | UniProtKB:Q96A29 | SLC35C1 |
| 135 | UniProtKB:Q96T58 | SPEN |
| 136 | UniProtKB:A8MQ27 | NEURL1B |
| 137 | UniProtKB:Q9NR61 | DLL4 |
| 138 | UniProtKB:Q9UHF1 | EGFL7 |
| 139 | UniProtKB:O60547 | GMDS |
| 140 | UniProtKB:Q9BQI9 | NRIP2 |
| 141 | UniProtKB:P12643 | BMP2 |
| 142 | UniProtKB:Q9Y6N7 | ROBO1 |
| 143 | UniProtKB:O95863 | SNAI1 |
| 144 | UniProtKB:O95858 | TSPAN15 |
| 145 | UniProtKB:O43439 | CBFA2T2 |
| 146 | UniProtKB:Q96LB3 | IFT74 |
| 147 | UniProtKB:P49810 | PSEN2 |
| 148 | UniProtKB:P49407 | ARRB1 |
| 149 | UniProtKB:Q9Y2G9 | SBNO2 |
| 150 | UniProtKB:Q9H3D4 | TP63 |
| 151 | UniProtKB:O95208 | EPN2 |
| 152 | UniProtKB:Q14469 | HES1 |
| 153 | UniProtKB:Q9ULJ6 | ZMIZ1 |
| 154 | UniProtKB:P29474 | NOS3 |
| 155 | UniProtKB:Q8NG11 | TSPAN14 |
| 156 | UniProtKB:Q9Y5A9 | YTHDF2 |
| 157 | UniProtKB:P25963 | NFKBIA |
| 158 | UniProtKB:Q8NCW6 | GALNT11 |
| 159 | UniProtKB:Q96FX8 | PERP |
| 160 | UniProtKB:P10721 | KIT |
| 161 | UniProtKB:P08727 | KRT19 |
| 162 | UniProtKB:Q9BUX1 | CHAC1 |
| 163 | UniProtKB:P50281 | MMP14 |
| 164 | UniProtKB:Q13207 | TBX2 |
| 165 | UniProtKB:P49768 | PSEN1 |
| 166 | UniProtKB:Q9UBV2 | SEL1L |
| 167 | UniProtKB:Q9UBP5 | HEY2 |
| 168 | UniProtKB:Q9Y219 | JAG2 |
| 169 | UniProtKB:Q8NES3 | LFNG |
| 170 | UniProtKB:Q96BI3 | APH1A |
| 171 | UniProtKB:P59773 | MINAR2 |
| 172 | UniProtKB:Q06330 | RBPJ |
| 173 | UniProtKB:Q9NVX2 | NLE1 |
| 174 | UniProtKB:Q9HCK4 | ROBO2 |
| 175 | UniProtKB:P55317 | FOXA1 |
| 176 | RNAcentral:URS0000435A77_9606 | URS0000435A77_9606 |
| 177 | RNAcentral:URS00001D69F6_9606 | URS00001D69F6_9606 |
| 178 | RNAcentral:URS00001D6BAE_9606 | URS00001D6BAE_9606 |
| 179 | UniProtKB:O95365 | ZBTB7A |
| 180 | UniProtKB:Q9UBC0 | ONECUT1 |
| 181 | UniProtKB:P40763 | STAT3 |
| 182 | UniProtKB:Q8NBL1 | POGLUT1 |
| 183 | UniProtKB:P15529 | CD46 |
| 184 | UniProtKB:O43903 | GAS2 |
| 185 | UniProtKB:Q9Y644 | RFNG |
| 186 | UniProtKB:Q9Y5J3 | HEY1 |
| 187 | UniProtKB:P17174 | GOT1 |
| 188 | UniProtKB:Q0VG99 | MESP2 |
| 189 | UniProtKB:Q8NFT8 | DNER |
| 190 | UniProtKB:Q96AX9 | MIB2 |
| 191 | UniProtKB:O60279 | SUSD5 |
| 192 | UniProtKB:Q9NQ87 | HEYL |
| 193 | UniProtKB:Q96KG7 | MEGF10 |
| 194 | UniProtKB:Q9UG01 | IFT172 |
| 195 | RNAcentral:URS0000424278_9606 | URS0000424278_9606 |

**Supplementary Table IV: A genes list of Notch Signaling Pathway (GO0007219)**

**
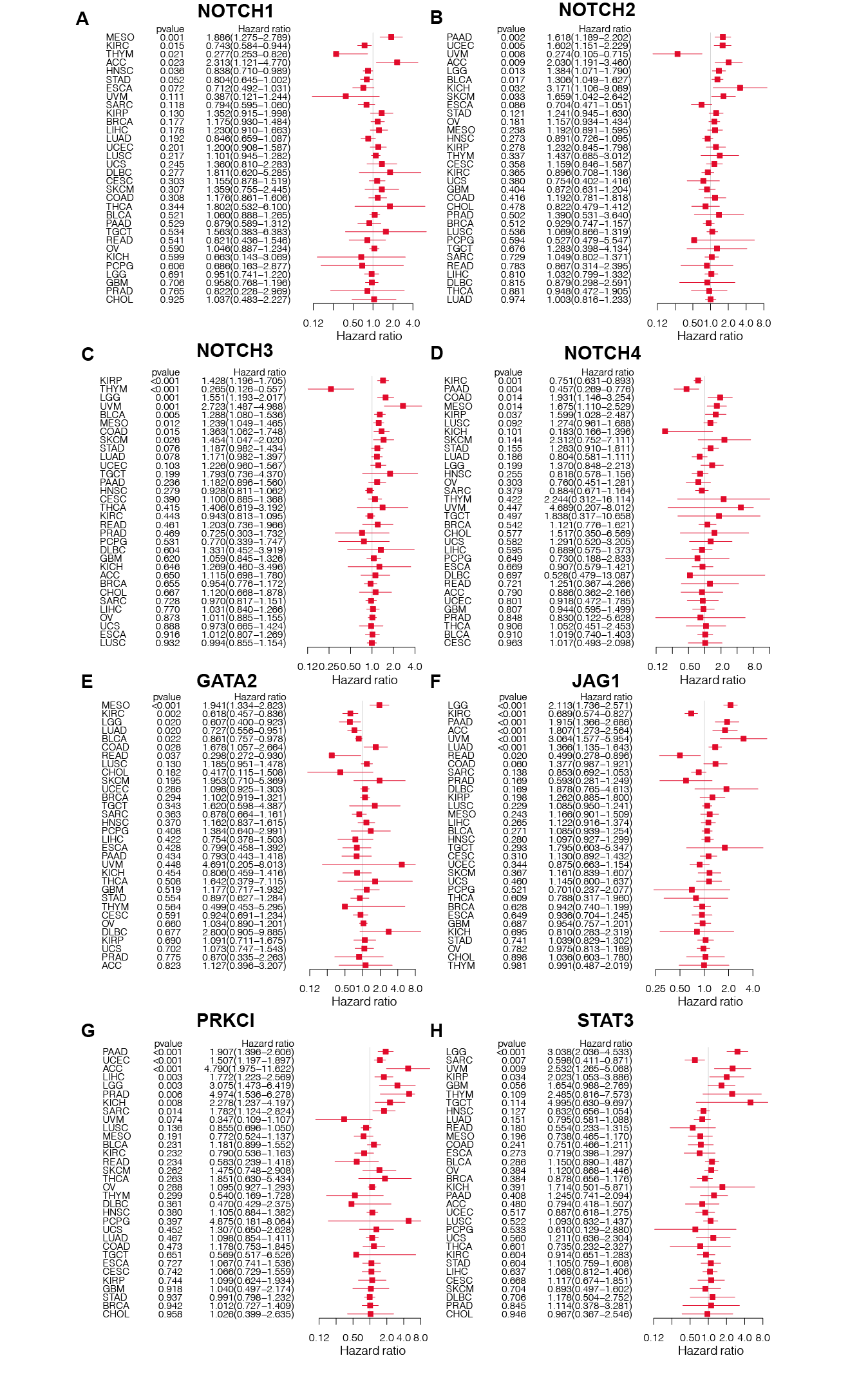
**

**Supplementary Fig. 9.** Cox regression analysis of sample genes in Notch signaling pathway.
